# Machine learning-based prediction model for teicoplanin plasma concentrations in adults with liver disease using real-world data

**DOI:** 10.3389/fphar.2025.1703976

**Published:** 2025-12-05

**Authors:** Fengbi Jian, Xiaodong Chen, Ming Wang, Zhihao Guo, Xuechun Li, Haobin Jian, Ronghong Ji, Liying Liang, Ze Yu, Yanfang Chen

**Affiliations:** 1 Department of Pharmacy, Guangzhou Eighth People’s Hospital, Guangzhou Medical University, Guangzhou, Guangdong, China; 2 Dalian Medicinovo Technology Co. Ltd, Dalian, China; 3 Beijing Medicinovo Technology Co. Ltd, Beijing, China

**Keywords:** teicoplanin, liver disease, machine learning, therapeutic drug monitoring, plasma concentration

## Abstract

**Objective:**

To construct a prediction model for teicoplanin (TEIC) plasma concentrations through machine learning and deep learning techniques in patients with liver disease using real-world clinical data.

**Methods:**

A retrospective study was conducted on patients who underwent TEIC therapeutic drug monitoring at a tertiary hospital in China (January 2019–March 2025). Dataset was split into training and test sets (8:2 ratio). Feature selection combined univariate analysis and algorithm importance ranking. Missing values were imputed using random forest (RF) model. Ten machine learning algorithms, such as RF, TransTab and light gradient boosting machine (LightGBM), were employed for model development, with predictive performance evaluated through 10-fold cross-validation on the training set. The optimal model was validated its predictive performance on the test set.

**Results:**

A total of 646 patients (689 TEIC concentrations) were eligible. Key variables were daily dose, hemoglobin (HGB), aspartate aminotransferase (AST), albumin (ALB), estimated glomerular filtration rate (eGFR), indirect bilirubin (IBIL), total bilirubin (TBIL), platelet count (PLT), urea and direct bilirubin (DBIL). LightGBM demonstrated superior predictive performance among ten algorithms, with a RMSE of 2.90, a R^2^ of 0.80, a MAE of 2.34, and 89.13% of accurate predictions within ±30% of observed concentrations on the independent test set. Daily dose, hemoglobin, and AST emerged as the most influential features.

**Conclusion:**

The LightGBM-based model integrating clinical covariates demonstrated robust predictive capability for TEIC plasma concentrations in liver disease. This tool provides real-world evidence to optimize TEIC dosing, advancing individualized treatment strategies to improve therapeutic outcomes in this population.

## 1 Introduction

Infections caused by methicillin-resistant *Staphylococcus aureus* (MRSA) and other Gram-positive bacteria are important adverse prognostic factors in adults with liver disease, noticeably increasing short-term mortality (Hung et al., 2024). Advanced liver disease weakens the immune function of patients, manifested clinically as increased susceptibility to infection, driven in part by systemic inflammation and intestinal flora dysbiosis (Agustín et al., 2022). Teicoplanin (TEIC) is a glycopeptide antibiotic commonly used to treat infections caused by Gram-positive bacteria (Hanai et al., 2022). Clinicians tend to choose TEIC for MRSA infections due to its comparable efficacy to vancomycin and notably lower incidence of adverse effects, especially nephrotoxicity and infusion reactions (Ju et al., 2024). Approximately 90% of TEIC is bound to serum albumin (ALB), and its antibacterial effect depends on the unbound fraction. Furthermore, multiple pathophysiological changes associated with liver disease can alter the pharmacokinetics of TEIC. As the primary site of ALB synthesis, hepatic dysfunction attenuates ALB production, which often results in hypoalbuminemia. Hypoalbuminemia markedly reduces the binding rate of drugs to ALB, thereby increasing their apparent volume of distribution (Vd) and clearance (CL). This results in subtherapeutic drug concentrations, impairing therapeutic efficacy (Tanaka, 2025). Severe liver disease is often complicated by acute kidney injury (AKI) and hepatorenal syndrome (HRS), inducing hemodynamic disorders and renal dysfunction, which in turn affects TEIC’s CL and increases the risk of toxic side effects (Téllez and Guerrero, 2022).

Previous population pharmacokinetic (PPK) studies have identified important covariates such as creatinine CL, ALB, and age in populations such as critically ill patients (Wang et al., 2023), elderly patients with pneumonia (Kang et al., 2023), and MRSA infection (Zhang et al., 2024), which can explain some of the individual variation. However, this method has limitations in adequately incorporating high-dimensional clinical variables (Hiroak et al., 2025), and a substantial portion of pharmacokinetic variability remains unexplained. To address these limitations and capture complex, nonlinear relations in clinical pharmacology data, machine learning has rapidly gained prominence in precision medicine (Mo et al., 2022; Huang S. et al., 2025). Machine learning models can effectively handle data and frequently demonstrate superior predictive performance compared to traditional PPK models (Kim et al., 2024; Chen et al., 2025; Hu et al., 2025; Huang Y. et al., 2025). Currently, research on individualized TEIC dosing in patients with liver disease is limited. Specifically, the application of machine learning for TEIC concentration prediction in this population is scarce, and existing drug labels and guidelines lack dose adjustment recommendations for these patients. This gap poses a significant clinical challenge, potentially compromising treatment outcomes. Therefore, there is an urgent need for research on machine learning guided individualized dosing strategies for TEIC in patients with liver disease to improve efficacy and safety. Kondo et al. (2025) established a 24-h loading dose regimen targeting a TEIC concentration of 15–30 μg/mL and identified four factors influencing the loading dose using a decision tree model. A study incorporated PPK parameters into a machine learning model, greatly improving the accuracy of predicting TEIC trough concentrations in critically ill patients (Ma et al., 2024). Given this context, this study aims to develop and validate a machine learning model using real-world clinical data to accurately predict TEIC plasma concentrations in adult patients with liver disease. The ultimate goal is to leverage this model to guide personalized dose adjustments, optimizing therapeutic efficacy while minimizing the risk of toxicity. We anticipate that this model will provide a robust foundation for implementing individualized TEIC dosing strategies in this complex patient population.

## 2 Methods

### 2.1 Participants and study design

**FIGURE 1:**
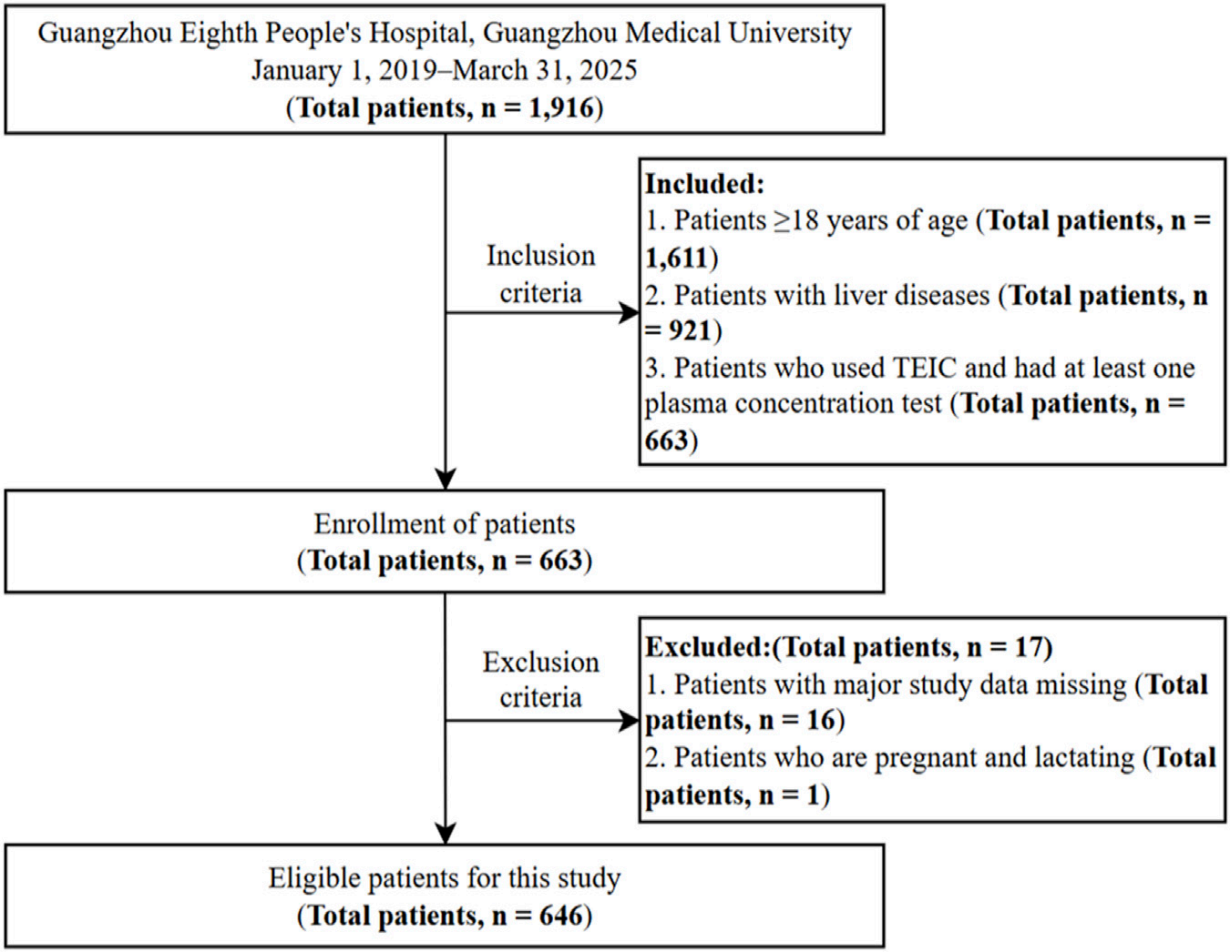
Patient selection flowchart showing inclusion and exclusion criteria. Note: Multiple TEIC administration and TDM records were collected during individual hospitalizations, resulting in 689 TDM records from 646 patients. Abbreviations: TEIC, teicoplanin; TDM, therapeutic drug monitoring.

This single-center, retrospective cohort study included adult patients with liver disease who received TEIC (Targocid^®^; Haizheng^®^) treatment at Guangzhou Eighth People’s Hospital, Guangzhou Medical University. Electronic health records, including hospital information system (HIS), laboratory information management system (LIS), and electronic medical records (EMRs), were systematically extracted from January 2019 to March 2025 to establish a comprehensive TEIC-related database. The inclusion criteria for this study were: (1) patients ≥18 years of age; (2) patients with liver diseases; (3) patients who used TEIC and had at least one plasma concentration test. The exclusion criteria for this study were: (1) patients with major study data missing; (2) patients who were pregnant and lactating. Patients who received a loading dose of 6–12 mg/kg every 12 h for 3 intravenous or intramuscular administrations, followed by maintenance dose ranging from 200 to 1,000 mg daily were included. The specific maintenance dose for each patient was individually tailored and adjusted by the clinician based on ongoing assessment of renal function and trough concentrations from therapeutic drug monitoring (TDM), aiming to achieve target therapeutic levels (>10 μg/mL). Figure 1 illustrates the workflow of sample selection. TEIC dosing regimens, administration intervals, and treatment duration were determined by attending physicians according to clinical judgment. Dose adjustments were guided by previously measured plasma concentrations, with blood samples collected 30 min before the third and fifth doses for therapeutic drug monitoring (TDM) (Kim et al., 2019).

The study protocol was approved by the Institutional Ethics Committee of Guangzhou Eighth People’s Hospital, Guangzhou Medical University (No. K202446348). All procedures adhered to the Declaration of Helsinki (1964) and its subsequent amendments. Patient data were de-identified prior to analysis in accordance with the Council for International Organizations of Medical Sciences/World Health Organization (CIOMS/WHO) International Ethical Guidelines for Health-related Research Involving Humans (2016). Approval for informed consent was waived due to the retrospective design of this study.

### 2.2 Blood sample collection and concentration determination

At least 3 days after the first dose of TEIC, serum samples were collected 30 min before the next dose. Serum TEIC concentrations were quantified using a high-performance liquid chromatography (HPLC) method. The linear range was 3.125–100 μg/mL (R^2^ = 0.9995). The lower limit of quantitation (LLOQ) was 3.125 μg/mL. Accuracy and precision were evaluated using LLOQ and quality control (QC) standards. The intra-day and inter-day precision were within 10%, and the accuracy for all LLOQ and QC standards was determined to be within 90%–110% (detailed validation parameters in Supplementary Table S1).

### 2.3 Outcome measures

According to the studies by Abdul-Aziz et al. (2020) and Pea (2020), as well as relevant clinical experience, the clinical outcomes were defined as follows: (1) for most Gram-positive bacterial infections, a TEIC trough concentration of at least 10 mg/L is recommended; (2) for endocarditis or other severe infections, the target trough concentration is 15–30 mg/L.

### 2.4 Data collection and preprocessing

Data from cases that achieved target therapeutic levels at the first monitoring timepoint were included for analysis. The dataset included the target variable (TDM value of TEIC), TEIC daily dose, demographic factors (age, gender, weight, height, body mass index [BMI]), disease histories (hypertension [HTN], diabetes, hyperlipidemia [HLP]), physiological and pathological factors (hepatitis disease, fatty liver disease [FLD], alcoholic liver disease [ALD], drug liver disease, liver cirrhosis, liver cancer, kidney disease, circulatory system disease, and gastrointestinal disease), decompensated liver cirrhosis, laboratory tests (ALB, total bilirubin [TBIL], hemoglobin [HGB], estimated glomerular filtration rate [eGFR], platelet count [PLT], indirect bilirubin [IBIL], aspartate aminotransferase [AST], urea, direct bilirubin [DBIL], low-density lipoprotein cholesterol [LDL-C], total cholesterol [TC], triglyceride [TG], alkaline phosphatase [ALP], gamma-glutamyl transferase [GGT], and high-density lipoprotein cholesterol [HDL-C]), concomitant medications (nephrotoxic ototoxic drugs and anticoagulant drugs). Variables with a missing data rate exceeding 50% or those exhibiting extreme class imbalance were excluded. The specific calculation formulas of the derived features are illustrated in Supplementary Appendix 1. During the research process, data standardization work was carried out simultaneously. After verification, the results after standardization processing showed no significant difference from the model results of the original data.

### 2.5 Feature engineering

The feature selection process involved two main steps. First, missing values for the remaining variables were imputed using a random forest (RF) algorithm. Second, univariate regression analysis was performed on the complete cohort to identify variables significantly associated with TEIC plasma concentrations (P < 0.05). To assess the robustness of our imputation strategy, we conducted a sensitivity analysis comparing six different imputation methods: RF, Bayesian, K-nearest neighbors (KNN), mean imputation, median imputation, and multiple imputation by chained equations (MICE). Model performance metrics [coefficient of determination (R^2^), the root mean square error (RMSE), and the mean absolute error (MAE)] were evaluated across all ten machine learning algorithms for each imputation method. RF imputation demonstrated consistently superior or comparable performance across the majority of model configurations and was therefore selected as the primary imputation strategy. Detailed sensitivity analysis results are provided in Supplementary Table S2.

### 2.6 Model establishment

**FIGURE 2:**
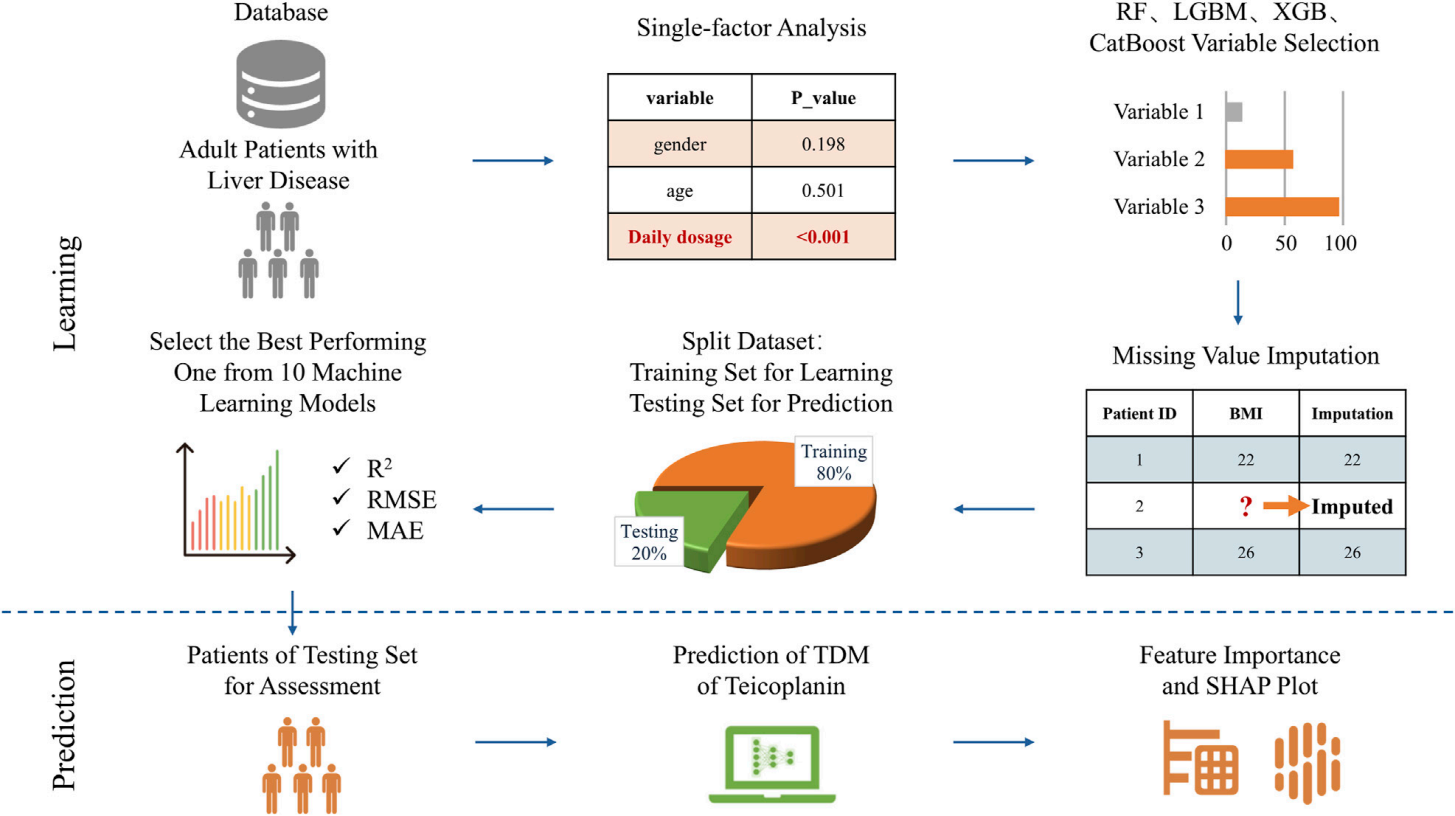
Machine learning workflow depicting the analysis process. Abbreviations: TDM, therapeutic drug monitoring; BMI, body mass index; RF, random forest; XGBoost, extreme gradient boosting; LightGBM, light gradient boosting machine; CatBoost, categorical boosting; RMSE, root mean square error; R^2^, coefficient of determination; MAE, mean absolute error.

The modeling workflow is depicted in Figure 2. The dataset was randomly partitioned into a training set and a test set, maintaining a ratio of 8:2. The training set was used to identify the best hyperparameters via a grid search algorithm and to build prediction models with 10-fold cross-validation. Within the training set, ten algorithms were used for modeling, including decision tree (DT), RF, extreme gradient boosting (XGBoost), light gradient boosting machine (LightGBM), categorical boosting (CatBoost), linearRegression, support vector machine (SVM), tabular prior-data fitted network (TabPFN), transferable tabular transformer (TransTab), and attentive interpretable tabular learning (TabNet). The principles of these algorithms are detailed in the Supplementary Appendix 2. In parallel, a traditional PPK model was developed using NONMEM software (version 7.3.0) to serve as a benchmark for comparison. The detailed PPK methodology, including model development, covariate screening, and evaluation strategies, is described in Supplementary Appendix 3. The corresponding results and model validation are presented in Supplementary Appendix 4. Full model parameterization data, encompassing both the hyperparameters selected during 10-fold cross-validation and the ten optimal model configurations, are detailed in the Supplementary Tables S3, S4. Model performance was evaluated using an independent test set. From the comparative performance analysis of all ten algorithms, the model demonstrating superior predictive metrics was selected for predicting TEIC plasma concentration. The final performance of each optimized model was evaluated on the test set using R^2^, RMSE, and MAE. The calculation formulas are as follows:

$$R^{2}=1-\frac{MSE\left(\hat{y},y\right)}{Var\left(y\right)}$$

$$\text{RMSE}=\sqrt{\frac{1}{n}{\sum_{i=1}^{n}\left(y_{i}-{\hat{y}}_{i}\right)}^{2}}$$

$$\text{MAE}=\frac{1}{n}\sum_{i=1}^{n}\left|\left(y_{i}-{\hat{y}}_{i}\right)\right|$$

The R^2^, which ranges from 0 to 1, quantifies the goodness-of-fit for the regression model, with higher values indicating a stronger fit. Conversely, lower values of RMSE and MAE reflect greater predictive accuracy. Optimal hyperparameter combinations were identified on the training set (80% of the data) by means of 10-fold cross-validation with random shuffling. These combinations were then evaluated on the independent 20% test set.

Furthermore, feature importance within the optimal model was systematically assessed, and importance scores were generated. SHAP (SHapley Additive exPlanations) values were subsequently applied to interpret the contribution of individual features to model predictions (Janssen et al., 2022).

### 2.7 Statistical analysis

Associations between baseline characteristics and TEIC plasma concentration were evaluated. For non-normally distributed variables, measurement data should be summarized using the median and interquartile range (IQR), whereas for normally distributed variables, the mean ± standard deviation is appropriate. Normality of data distributions was assessed using the Kolmogorov-Smirnov test. Normality of data distributions was assessed using the Kolmogorov-Smirnov test. Based on distributional characteristics, appropriate statistical tests were applied: Student’s t-test for analyzing relationships between the target variable and continuous/binary variables in normally distributed data; the Spearman rank correlation test for assessing significance between the target variable and continuous variables in non-normally distributed data; and the Mann-Whitney U test for evaluating associations between the target variable and binary variables in non-normally distributed data. Continuous variables are presented as median (IQR), while categorical variables are expressed as frequency (percentage). All statistical analyses were performed using SPSS software (version 25.0; IBM Corp.) and Python (version 3.7).

## 3 Results

### 3.1 Baseline patient characteristics

The workflow of sample selection is shown in Figure 1. A total of 1,916 patients were hospitalized at Guangzhou Eighth People’s Hospital, Guangzhou Medical University between January 2019 and March 2025. After data screening, 689 TDM records from 646 patients were included for this study. The demographic and clinical characteristics of the 646 eligible patients are presented in Table 1. The median (IQR) age of the patients was 60.00 (50.00∼72.00) years old, with a median (IQR) weight of 60.00 (51.00∼67.93) kg, a median (IQR) BMI of 21.45 (19.71∼24.82) kg/m^2^, and a median (IQR) height of 165.75 (160.00∼170.00) cm. Males accounted for 73.00%. The TEIC data revealed a median (IQR) TDM value of 15.29 (11.95∼19.36) µg/mL, and a median (IQR) daily dose of 0.40 (0.40∼0.60) g. In the disease histories, HTN, diabetes and HLP accounted for 33.09%, 26.71% and 10.01%, respectively. Among the physiological and pathological factors, circulatory system disease, kidney disease, gastrointestinal disease, hepatitis disease, liver cirrhosis, FLD, liver cancer, ALD and drug liver disease accounted for 70.25%, 69.23%, 40.93%, 28.16%, 20.61%, 13.64%, 10.60%, 2.03% and 1.16%, respectively. Decompensated liver cirrhosis was present in 14.51% of patients. In concomitant medications, nephrotoxic ototoxic drugs and anticoagulant drugs accounted for 11.90% and 1.16%, respectively. Furthermore, the 689 TDM records was randomly divided into training (n = 551, 80%) and test sets (n = 138, 20%).

**TABLE 1:** Demographic and characteristic statistical description.

Category	Variable	Median (IQR) | n (%)	Miss rate
Target variable	TDM, μg/mL	15.29 (11.95∼19.36)	0.00%
TEIC daily dose	Daily dose, g	0.40 (0.40∼0.60)	0.00%
Demographic information	Sex		0.00%
	Male	503 (73.00%)	
	Female	186 (27.00%)	
	Age, years	60.00 (50.00∼72.00)	0.00%
	Weight, kg	60.00 (51.00∼67.93)	30.33%
	Height, cm	165.75 (160.00∼170.00)	24.24%
	BMI, kg/m^2^	21.45 (19.71∼24.82)	34.25%
Disease history	HTN	228 (33.09%)	0.00%
	Diabetes	184 (26.71%)	0.00%
	HLP	69 (10.01%)	0.00%
Physiological and pathological factor	Hepatitis disease	194 (28.16%)	0.00%
	FLD	94 (13.64%)	0.00%
	ALD	14 (2.03%)	0.00%
	Drug liver disease	8 (1.16%)	0.00%
	Liver cirrhosis	142 (20.61%)	0.00%
	Liver cancer	73 (10.60%)	0.00%
	Kidney disease	477 (69.23%)	0.00%
	Circulatory system disease	484 (70.25%)	0.00%
	Gastrointestinal disease	282 (40.93%)	0.00%
Decompensated liver clirrhosis	Decompensated liver clirrhosis	100 (14.51%)	0.00%
Laboratory test	CRP, mg/L	42.46 (18.09∼88.76)	0.58%
	ALT, U/L	26.00 (11.62∼54.00)	1.89%
	NEU, %	80.30 (70.10∼87.20)	0.00%
	LDL-C, mmol/L	31.00 (2.01∼63.00)	70.10%
	AST, U/L	43.00 (25.00∼79.00)	1.60%
	Urea, mmol/L	11.15 (6.10∼19.70)	6.82%
	TC, mmol/L	94.00 (3.48∼124.25)	69.81%
	TBIL, μmol/L	25.20 (10.60∼116.75)	2.03%
	TP, g/L	59.40 (53.32∼66.38)	2.18%
	TG, mmol/L	144.00 (4.53∼286.00)	69.81%
	WBC, 10^9^/L	9.22 (5.54∼13.85)	0.00%
	ALB, g/L	33.60 (30.20∼37.10)	1.89%
	DBIL, μmol/L	18.00 (6.00∼96.45)	2.03%
	ALP, U/L	142.00 (111.00∼206.50)	62.55%
	eGFR, mL/min/1.73m^2^	54.65 (23.89∼102.31)	34.83%
	Cys-C, mg/L	2.17 (1.40∼3.51)	49.78%
	PLT, 10^9^/L	124.00 (60.00∼239.00)	0.00%
	Scr, μmol/L	98.45 (59.85∼199.35)	3.05%
	GGT, U/L	85.00 (42.00∼190.00)	62.55%
	Glu, mmol/L	6.83 (4.72∼10.03)	47.75%
	HGB, g/L	76.00 (64.00∼95.00)	0.00%
	IBIL, μmol/L	7.50 (4.10∼21.85)	2.03%
	PCT, ng/mL	0.83 (0.32∼2.58)	1.02%
	HDL-C, mmol/L	13.00 (0.72∼23.75)	70.10%
Concomitant medication	Nephrotoxic ototoxic drugs, %	82 (11.90%)	0.00%
	Anticoagulant drugs, %	8 (1.16%)	0.00%

Abbreviations: TDM, therapeutic drug monitoring; BMI, body mass index; HTN, hypertension; HLP, hyperlipidemia; FLD, fatty liver disease; ALD, alcoholic liver disease; CRP, C-reactive protein; ALT, alanine aminotransferase; NEU, neutrophil ratio; LDL-C, low-density lipoprotein cholesterol; AST, aspartate aminotransferase; TC, total cholesterol; TBIL, total bilirubin; TP, total protein; TG, triglyceride; WBC, white blood count; ALB, albumin; DBIL, direct bilirubin; ALP, alkaline phosphatase; eGFR, estimated glomerular filtration rate; Cys-C, cystatin-C; PLT, platelet count; Scr, serum creatinine; GGT, gamma-glutamyl transferase; Glu, glucose; HGB, hemoglobin; IBIL, indirect bilirubin; PCT, procalcitonin; HDL-C, high-density lipoprotein cholesterol.

### 3.2 Variable selection

**FIGURE 3:**
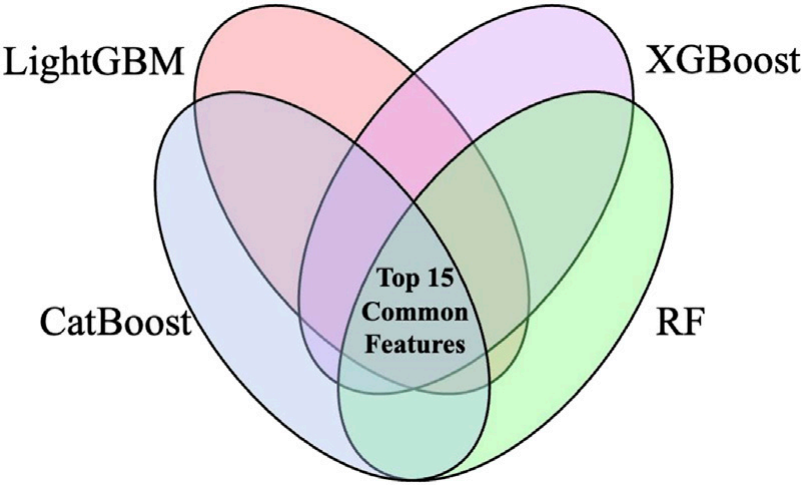
Feature selection workflow based on feature importance rankings from four machine learning algorithms. Abbreviations: RF, random forest; XGBoost, extreme gradient boosting; LightGBM, light gradient boosting machine; CatBoost, categorical boosting.

A total of 45 variables were initially recorded (Table 1). Subsequently, some variables were excluded, including LDL-C, TC, TG, ALP, GGT, and HDL-C which had missing rates greater than 50%, while ALD, drug liver disease, and anticoagulant drugs which presented highly imbalanced categorical samples. Afterwards, variables were subjected to univariate analysis, of which 15 variables presented P < 0.05 (Table 2). Four machine learning models - CatBoost, LightGBM, XGBoost, and RF - were employed to rank feature importance. By performing intersection operations on the top 15 most important features from each model, a final set of 10 core variables was determined, including: ALB, TBIL, HGB, eGFR, PLT, IBIL, AST, urea, daily dose, and DBIL (Figure 3; detailed rankings in Supplementary Table S5).

**TABLE 2:** Significance analysis of TEIC TDM and individual variables.

Variable	*P* value	Variable	*P* value
Daily dose	<0.001	AST	<0.001
Sex	0.198	Urea	0.022
Age	0.501	TC	0.909
Weight	0.069	TBIL	0.006
Height	0.384	TP	0.214
BMI	0.035	TG	0.212
HTN	0.028	WBC	0.357
Diabetes disease	0.057	ALB	<0.001
HLP	0.058	DBIL	0.003
Hepatitis disease	0.009	ALP	0.207
FLD	0.403	eGFR	0.007
ALD	<0.001	Cys-C	0.668
Drug liver disease	0.323	PLT	0.032
Liver cirrhosis	0.005	Scr	0.293
Liver cancer	0.865	GGT	0.663
Kidney disease	0.071	Glu	0.848
Circulatory system disease	0.001	HGB	0.036
Gastrointestinal disease	0.272	IBIL	0.009
Decompensated liver cirrhosis	0.066	PCT	0.559
CRP	0.574	HDL-C	0.279
ALT	0.196	Nephrotoxic ototoxic drugs	0.070
NEU%	0.898	Anticoagulant drugs	0.251
LDL-C	0.180		

Abbreviations: TDM, therapeutic drug monitoring; BMI, body mass index; HTN, hypertension; HLP, hyperlipidemia; FLD, fatty liver disease; ALD, alcoholic liver disease; CRP, C-reactive protein; ALT, alanine aminotransferase; NEU, neutrophil ratio; LDL-C, low-density lipoprotein cholesterol; AST, aspartate aminotransferase; TC, total cholesterol; TBIL, total bilirubin; TP, total protein; TG, triglyceride; WBC, white blood count; ALB, albumin; DBIL, direct bilirubin; ALP, alkaline phosphatase; eGFR, estimated glomerular filtration rate; Cys-C, cystatin-C; PLT, platelet count; Scr, serum creatinine; GGT, gamma-glutamyl transferase; Glu, glucose; HGB, hemoglobin; IBIL, indirect bilirubin; PCT, procalcitonin; HDL-C, high-density lipoprotein cholesterol.

After initial variable selection, as shown in Supplementary Table S6, no significant differences (P > 0.05) were observed in baseline characteristics between the two sets, including TEIC TDM (training: 15.29 [IQR 11.83∼19.37] µg/mL vs. test: 15.28 [12.24∼19.10] µg/mL; P = 0.734), daily dose (0.40 [0.40∼0.60] g; P = 0.773), and key laboratory parameters (such as AST, urea, TBIL, and ALB), confirming partition validity.

### 3.3 Model performance

Ten machine learning and deep learning models were developed using the selected variables to predict the TEIC plasma concentration. The 10-fold cross-validation results on the training set are presented in Table 3. The results demonstrate that the LightGBM model achieves a mean R^2^ value of 0.69 with a low standard deviation of 0.03, indicating greater stability and better fitting performance compared to other models.

**TABLE 3:** The ten-fold cross-validation results of the model on the training set (mean ± std).

Algorithm	RMSE	R^2^	MAE
LightGBM	3.35 ± 0.37	0.69 ± 0.03	2.69 ± 0.27
CatBoost	3.58 ± 0.53	0.65 ± 0.03	2.83 ± 0.33
XGBoost	3.82 ± 0.62	0.60 ± 0.05	2.98 ± 0.39
LinearRegression	3.56 ± 0.40	0.65 ± 0.04	2.85 ± 0.28
SVM	4.01 ± 0.55	0.55 ± 0.05	3.08 ± 0.37
TabPFN	3.37 ± 0.34	0.68 ± 0.04	2.72 ± 0.27
TransTab	3.67 ± 0.43	0.62 ± 0.05	2.83 ± 0.23
TabNet	3.72 ± 0.43	0.60 ± 0.09	2.90 ± 0.27
DT	3.43 ± 0.34	0.67 ± 0.04	2.81 ± 0.29
RF	3.92 ± 0.59	0.58 ± 0.02	3.01 ± 0.36

Abbreviations: RMSE, root mean square error; R^2^.

coefficient of determination; MAE, mean absolute error; DT, decision tree; RF, random forest; XGBoost, extreme gradient boosting; LightGBM, light gradient boosting machine; CatBoost, categorical boosting; SVM, support vector machine; TabPFN, tabular prior-data fitted network; TabNet, attentive interpretable tabular learning; TransTab, transferable tabular transformer.

**FIGURE 4:**
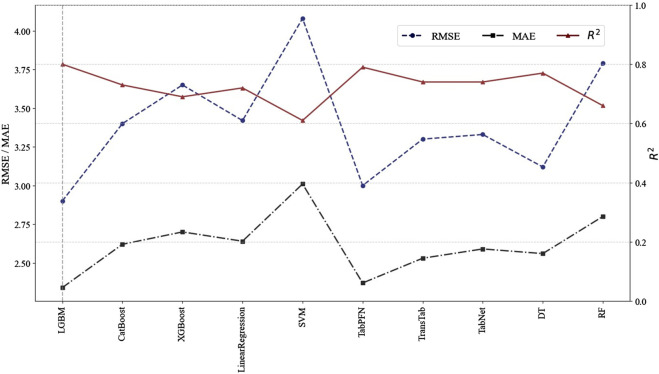
Model performance comparison on the test set. Note: The figure displays the RMSE (mean ± std), R^2^ (mean ± std), and MAE (mean ± std) calculated for the test set. The left y-axis indicates the scales of RMSE and MAE, whereas the right y-axis indicates the scale of R^2^. Abbreviations: RMSE, root mean square error; R^2^, coefficient of determination; MAE, mean absolute error; DT, decision tree; RF, random forest; XGBoost, extreme gradient boosting; LightGBM, light gradient boosting machine; CatBoost, categorical boosting; SVM, support vector machine; TabPFN, tabular prior-data fitted network; TabNet, attentive interpretable tabular learning; TransTab, transferable tabular transformer.

The validation results of each model on the test set are shown in Figure 4, with detailed data provided in Supplementary Table S7. Among the ten algorithms evaluated, LightGBM exhibited the optimal performance. It achieved a RMSE of 2.90, a R^2^ of 0.80, and a MAE of 2.34. As shown in Table 4, LightGBM also achieved the highest accuracy, with an accuracy rate of 89.13% for predictions falling within ±30% of the actual values. Based on the overall assessment of all evaluation metrics, LightGBM demonstrated superior predictive capability. Therefore, LightGBM was selected to develop the final model.

**TABLE 4:** The comparison of prediction accuracy results of the models on the test set.

Algorithm	Accuracy within ± 30%	Accuracy within ± 40%	Accuracy within ± 50%
LightGBM	89.13	97.10	98.55
CatBoost	83.33	93.48	96.38
XGBoost	81.16	89.86	95.65
LinearRegression	84.78	92.75	98.55
SVM	81.16	92.75	96.38
TabPFN	87.68	96.38	98.55
TransTab	85.51	94.93	97.83
TabNet	86.96	95.65	96.38
DT	83.33	97.10	97.83
RF	85.51	92.75	94.20

Abbreviations: DT, decision tree; RF, random forest; XGBoost, extreme gradient boosting; LightGBM, light gradient boosting machine; CatBoost, categorical boosting; SVM, support vector machine; TabPFN, tabular prior-data fitted network; TabNet, attentive interpretable tabular learning; TransTab, transferable tabular transformer.

Accuracy metrics represent the percentage of model predictions on the test set that fall within the specified error margin (±30%, ±40%, or ±50%) of the observed teicoplanin plasma concentrations.

The predictive performance of all models was visualized through scatter plots of predicted versus observed TEIC plasma concentrations (Supplementary Figure S1). LightGBM showed the strongest linear alignment (R^2^ = 0.80). Supplementary Figure S2 displays the distribution of prediction errors. LightGBM achieved the most consistent error profile, with 89.13% of predictions falling within ±30% of observed values, supporting its clinical reliability.

### 3.4 Comparison with PPK model

To provide a comparative benchmark, a traditional PPK model was also established. The PPK analysis identified urea as a notable covariate affecting TEIC CL (Supplementary Appendix 4). The PPK model (Supplementary Appendix 4) yielded a R^2^ of 0.68, a RMSE of 22.60, a MAE of 16.11, and a P30 of 53.33%. In addition, a visual predictive check (VPC) was performed to evaluate the time-dependent predictive performance of the final PPK model. The observed TEIC concentrations over time are shown in Supplementary Figure S3. The concentration fluctuates greatly from 0 to 200 h, and enters a steady state from 200 to 1,000 h. The red and blue shadows (confidence intervals) basically cover the red observation percentile line, indicating that the model can accurately fit the concentration distribution from the loading period to the steady-state period, verifying the effective integration of time dimension information in the model.

### 3.5 Model interpretation

The prediction model for TEIC plasma concentration, based on the LightGBM model, calculates the importance score of each variable. A higher importance score indicates a greater influence of that variable on the plasma concentration prediction. As shown in Table 5, the three most influential features in the LightGBM model for predicting TEIC plasma concentration were daily dose, HGB, and AST, in a descending order of impacts.

**TABLE 5:** Variable importance scores based on the LightGBM model.

Feature name	Feature importance
Daily dose	649
HGB	229
AST	137
eGFR	131
ALB	119
IBIL	92
Urea	87
TBIL	82
PLT	70
DBIL	15

Abbreviations: LightGBM, light gradient boosting machine; HGB, hemoglobin; AST, aspartate aminotransferase; eGFR, estimated glomerular filtration rate; ALB, albumin; IBIL, indirect bilirubin; TBIL, total bilirubin; PLT, platelet count; DBIL, direct bilirubin.

**FIGURE 5:**
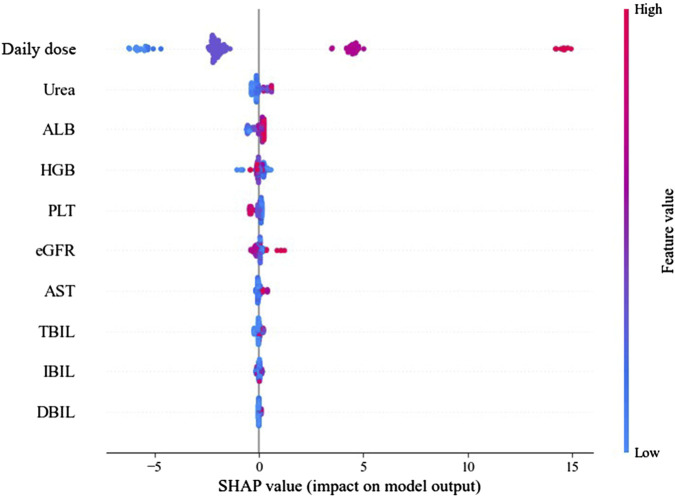
SHAP plot based on the LightGBM model. Each point represents a patient sample, with color gradient indicating covariate values (red: high; blue: low). Positive SHAP values indicate features that increase predicted TEIC plasma concentration, while negative values indicate features that decrease it. Abbreviations: LightGBM, light gradient boosting machine; HGB, hemoglobin; AST, aspartate aminotransferase; eGFR, estimated glomerular filtration rate; ALB, albumin; IBIL, indirect bilirubin; TBIL, total bilirubin; PLT, platelet count; DBIL, direct bilirubin.

Based on the SHAP plots (Figure 5 and the Supplementary Figure S4), both the positive and negative correlations between variables and the plasma concentration prediction model, as well as the strength of these correlations, are illustrated. Each row in the following figure represents an individual variable, while the horizontal axis corresponds to the SHAP value. Each data point represents a sample; the redder the color, the higher the feature value, whereas the bluer the color, the lower the feature value. If most red points are concentrated in the region where the SHAP value on the horizontal axis is greater than zero, it indicates a positive correlation between the variable and the target variable; conversely, a concentration of blue points in that region suggests a negative correlation. It can be concluded that daily dose, urea, and ALB exhibit a positive association with TEIC plasma concentration.

To quantify the impact of continuous variables on TEIC concentration, predicted concentrations were calculated for each quartile of the nine continuous features using the LightGBM model. As shown in Supplementary Figure S5, daily dose, urea, and ALB demonstrated positive associations with TEIC plasma concentration, consistent with SHAP analysis results.

## 4 Discussion

Based on real-world data, this study developed an individualized prediction model for TEIC plasma concentrations in adult patients with liver disease. The discovery of HGB, AST, IBIL, TBIL, and DBIL as new factors influencing TEIC plasma concentrations in patients with liver disease lays an important foundation for the development of individualized clinical dosing strategies. The core value of this study lies in filling the real-world evidence gap in TEIC dose adjustment for patients with liver disease. Although current guidelines offer recommendations for dose regimens in patients with renal insufficiency, severe infection, or continuous veno-venous hemodialysis, they lack specific guidance for patients with liver disease (Hanai et al., 2022; Choi et al., 2023). In this study, we comprehensively analyzed real-world clinical data from a single center, including a large sample of TEIC plasma concentrations (n = 689). On the basis of univariate analysis, we further employed machine learning methods, which revealed that multiple factors influence TEIC concentrations in liver disease patients. After identifying these key influencing factors, the constructed LightGBM model achieved high-precision prediction (R^2^ = 0.80, accuracy within ±30% = 89.13% in the test set), providing valuable guidance for dose adjustment of TEIC in clinical practice. A key finding of this study is the marked difference in predictive performance between the machine learning and traditional PPK approaches. When evaluated on the current dataset, the PPK model, which identified urea as a notable covariate, achieved moderate predictive performance (R^2^ = 0.68, RMSE = 22.60, MAE = 16.11, accuracy within ±30% = 53.33%). In contrast, the LightGBM model, which integrated 10 clinical variables, yielded substantially better performance (R^2^ = 0.80, RMSE = 2.90, MAE = 2.34, accuracy within ±30% = 89.13%). This enhanced accuracy is likely attributable to the machine learning algorithm’s ability to capture complex, nonlinear interactions among renal, hepatic, and hematological parameters (Roscher et al., 2020). These results suggest that while PPK modeling provides a valuable mechanistic framework (Mould and Upton, 2012), machine learning is better suited for developing predictive tools in clinically complex and heterogeneous patient populations, such as those with liver disease.

Key feature analysis provides a deep perspective for understanding the pharmacokinetics of TEIC in patients with liver disease. Our analysis identified ten key feature variables for this model. Consistent with previous literature, our findings confirm the significance of daily dose, eGFR, ALB, urea, and PLT. More importantly, we screened out five variables—HGB, AST, IBIL, TBIL, and DBIL—that have not been previously reported in TEIC model research (Ma et al., 2022; 2024; Kondo et al., 2025). Among all variables, daily dose as the most significant influencing factor (importance score = 649), is consistent with the fundamental pharmacokinetic principle of the dose-concentration relationship, thereby validating the rationality of the model. Furthermore, the high importance of HGB and AST highlights specific physiological and pathological mechanisms in this patient population. HGB levels may influence the apparent Vd of the drug by reflecting the patient’s anemic status (where anemia may be accompanied by fluid retention) and circulating blood volume (such as chronic blood loss caused by portal hypertension in liver cirrhosis). This leads to a decrease in blood drug concentration, thus requiring a higher TEIC dose to achieve effective concentrations (Tanaka, 2025). Meanwhile, elevated AST, a sensitive marker of hepatic dysfunction, suggests hepatocellular injury and impaired liver function. This impairment may reduce the activity of hepatic drug-metabolizing enzymes and delay the excretion of drug metabolites, consequently affecting drug CL efficiency (Sookoian and Pirola, 2015). A likely reason why ALT was not selected as a key predictor is that in advanced liver disease, particularly with malnutrition or alcohol-related disease, pyridoxal-5′-phosphate can deficiency disproportionately reduces ALT activity relative to AST, leading to lower measured ALT levels (Domanski and Harrison, 2013). Additionally, reduced functional hepatocyte mass in advanced cirrhosis can result in normal or even low aminotransferase levels despite severe disease (Youssef and Wu, 2024).

The high importance of HGB and AST reflects key physiological processes in cirrhotic patients. Low HGB indicates anemia, which in liver disease is often accompanied by plasma volume expansion caused by splanchnic vasodilation and activation of the renin-angiotensin-aldosterone system, increasing the Vd of hydrophilic drugs such as TEIC (Pimpin et al., 2018). Chronic blood loss from portal hypertension (such as esophageal varices) and impaired hepatic synthesis further alter hemodynamics and inflammation, affecting drug exposure (Garcia-Tsao and Bosch, 2010; Qamar and Grace, 2009). These mechanisms may explain why reduced HGB is associated with lower TEIC concentrations.

Elevated AST, a marker of hepatocellular injury, may influence drug disposition through several pathways. Damage to hepatocytes and hepatic sinusoids can impair drug uptake and biliary excretion (Rostami-Hodjegan and Tucker, 2004), while mitochondrial dysfunction-as reflected by elevated AST levels—may impair cellular metabolic capacity (Sookoian and Pirola, 2015). Additionally, inflammation-mediated downregulation of drug-metabolizing enzymes and transporters further contributes to reduced clearance (Morgan et al., 2008). Consequently, TEIC CL may decrease with rising AST levels. The predominance of AST over ALT as a predictor is consistent with the higher AST/ALT ratio observed in advanced or alcohol-related liver injury (Nyblom et al., 2004; Giannini et al., 2005).

Abnormalities in bilirubin indices (TBIL, IBIL, DBIL) reflect impaired hepatobiliary excretion (Meijer et al., 1997), which can indirectly affect drug CL in patients with liver disease. Cheng et al. reported that DBIL showed a significant effect on the trough concentration of TEIC (Cheng et al., 2025). This effect may be explained by the fact that bilirubin is predominantly bound to plasma proteins (up to 90%). Elevated bilirubin levels may alter drug-protein interactions. Bilirubin binds tightly to ALB at Sudlow site I, the major binding region for many acidic drugs (Brodersen, 1979). At high concentrations, bilirubin competitively displaces albumin-bound ligands, thereby increasing the unbound fraction (Ehrnebo et al., 1971; Jacobsen and Wennberg, 1974). In patients with hepatic dysfunction, such displacement may enhance renal CL and tissue distribution of highly bound compounds such as TEIC, resulting in lower total drug concentrations (Verbeeck, 2008).

To address potential collinearity between bilirubin indices and ALB, we note that the LightGBM algorithm inherently mitigates the effects of multicollinearity through tree-based feature selection and regularization (Ke et al., 2017). Moreover, bilirubin indices capture aspects of hepatic physiology different from those reflected by albumin, including bilirubin-induced alterations in protein binding and pharmacokinetics (Rodighiero, 1999). Therefore, collinearity is unlikely to have materially influenced our results, and bilirubin indices likely provide independent predictive information beyond ALB.

Both eGFR and urea reflect the important role of the kidneys in the pharmacokinetic process of TEIC. Several PPK models have shown that eGFR is strongly correlated with the unbound TEIC and influences the inter-individual variability in CL, making eGFR an important factor in determining the optimal trough concentration of TEIC (Fu et al., 2022; Wang et al., 2023). For example, patients with reduced renal function were able to reach the target trough concentration (15 μg/mL) with a shorter duration of loading dose treatment (1 day) compared to those with normal renal function, who required a longer loading period (2 days) (Takechi et al., 2017). Besides, elevated urea level often indicates impaired renal function. In patients with liver disease, especially those with advanced cirrhosis, renal dysfunction (such as HRS) is common (Téllez and Guerrero, 2022). TEIC is mainly excreted through the kidneys, and decreased renal function may lead to a reduction in its clearance rate. Therefore, it is essential for clinicians to consider patients’ eGFR and urea levels when adjusting the dosing regimen, in order to maintain effective therapeutic concentrations of TEIC while minimizing the risk of toxicity due to drug accumulation.

Moreover, this study clearly demonstrates the significant impact of serum ALB levels on TEIC pharmacokinetics in patients with liver disease. TEIC is a highly protein-bound drug, with approximately 90% of the compound bound to plasma albumin (Zhang et al., 2024). As an important facility for ALB synthesis, the liver will have an imbalance of ALB metabolism in the pathological state (Domenicali et al., 2014): decreased synthesis due to impaired hepatocyte function and accelerated consumption and decomposition due to the inflammation, jointly leads to a reduced level of ALB. Variations in serum ALB levels can therefore markedly influence both the CL and Vd of TEIC. Reduced ALB levels lead to an increased proportion of unbound TEIC, and only the unbound form is pharmacologically active and can be cleared (Brink et al., 2015; Zhang et al., 2024). Hepatic dysfunction also indirectly affects TEIC CL. Severe hepatic dysfunction leading to HRS and AKI affects kidney function, thereby impacting TEIC CL (Cullaro et al., 2022; Pose et al., 2024). Barbot A et al. found a negative relationship between serum ALB levels and the total apparent CL of TEIC (Barbot et al., 2003). It verifies the rationality that ALB levels were positively associated with TEIC TDM values. Our results are in agreement with previous research, such as studies by Zhang et al. (2024) and Soy et al. (2006), which found that patients with lower ALB levels, such as those with liver cirrhosis, benefit more from adjusted TEIC dosing strategies. Our findings suggest that the increase in the free fraction of TEIC due to decreased ALB is a key driving factor in patients with liver disease, further supporting the model’s ability to capture pathophysiological mechanisms.

In addition, thrombocytopenia (low PLT counts) is common in liver disease, and the possible mechanisms include hypersplenism in cirrhosis, reduced thrombopoietin production by the liver, or immunological removal of platelets from the circulation (Peck Radosavljevic, 2017). Severe thrombocytopenia often correlates with advanced liver dysfunction, which may be accompanied by impaired hepatic metabolism or biliary excretion of drugs, thereby indirectly reducing drug CL. Notably, a trough concentration above 40 μg/mL has been identified as a risk factor for TEIC-induced thrombocytopenia (Kasai et al., 2018). In brief, to minimize the risk of drug-induced adverse effects, it is important to closely monitor platelet counts when determining the dosage regimen of TEIC in patients with liver disease.

In terms of model performance, the LightGBM algorithm performed the best among ten machine learning and deep learning models in this study. LightGBM is an ensemble learning method based on the boosting strategy and an improved version of the GBDT framework, incorporating fast, distributed, and high-performance characteristics (Ke et al., 2017). The key advantage of machine learning and deep learning lies in their capacity to capture nonlinear pharmacokinetic relationships. This is particularly critical in liver disease patients, whose drug metabolism may be affected by complex interactions between hepatic and renal impairments (e.g., HRS). Compared to traditional linear models (such as LinearRegression), machine learning-based approaches typically provide superior predictive accuracy, as conventional models often fail to adequately represent the intricate relationships present in clinical data (Ota and Yamashita, 2022). Recently, machine learning techniques have been increasingly applied for predicting drug concentrations and optimizing dosing regimens. Representative examples include the use of XGBoost for predicting venlafaxine concentration and LightGBM for estimating warfarin maintenance doses (Liu et al., 2021; Chang et al., 2024).

Currently, both clinical guidelines and previous research on the use of TEIC in patients with liver disease are limited. Most studies and recommendations have primarily focused on the impact of renal function on optimal concentration and dose adjustment, while ignoring the impact of liver disease on TEIC metabolism and efficacy (Hanai et al., 2021; Xu et al., 2022). Several PPK models have been applied to analyze TEIC dosing regimens, but they are typically targeted at critically ill patients (Chen et al., 2023; Kang et al., 2023; Ma et al., 2024). Therefore, we conducted a comprehensive analysis using a large cohort of liver disease patients, addressing the current gap in TEIC dosing recommendations for this population. In addition, compared to the PPK model that assumes specific mathematical equation, machine learning does not pre-set strict physiological models. Machine learning algorithms can learn various patterns and associations from data, more effectively capturing complex nonlinear relationships in the real world. In this study, the machine learning-based findings establish a crucial evidence base to help clinicians optimize TEIC therapy in hepatic impairment, thereby improving treatment efficacy and safety.

While our LightGBM model achieved superior predictive performance (R^2^ = 0.80, accuracy within ±30% = 89.13%) compared to the PPK model (R^2^ = 0.68, accuracy within ±30% = 53.33%), the trade-off between predictive accuracy and interpretability warrants consideration. PPK models provide mechanistic frameworks enabling simulation of dosing regimens and prediction of concentration-time profiles across clinical scenarios, which directly inform individualized TEIC therapy (Mould and Upton, 2012; Abdul-Aziz et al., 2020; Marshall et al., 2016). Conversely, machine learning models excel in capturing complex nonlinear relationships but function as “black boxes” with limited mechanistic transparency, despite advances in explainable AI such as SHAP analysis (Ribeiro et al., 2016; Janssen et al., 2022). An optimal approach may involve integrating both methodologies-using PPK models for physiological simulation and machine learning for precision enhancement-potentially through hybrid frameworks that leverage complementary strengths (Rackauckas et al., 2021).

The PPK model constructed in this study has poor fitting performance and inherent flaws, resulting in RMSE and MAE being much higher than those of LightGBM. First, the one-compartment model selected for the PPK model is relatively simplified, describing the pharmacokinetic process using only two parameters (CL/F and V/F). However, patients with liver disease have complex pathological conditions, and their actual pharmacokinetic process is closer to a two-compartment model. Unfortunately, the existing data in this study lack the distribution phase concentration data and sufficient dynamic information, which are necessary for a two-compartment model, and can only support the fitting of a one-compartment model. The TEIC blood samples in the paper are steady-state trough concentrations, which reflect the steady-state level of the drug elimination phase, and lack distribution phase concentration data in the early post-administration period (such as 0.5h, 1h, 2 h after administration). Additionally, as this study used a retrospective dataset, sufficient dynamic change information was not obtained, which may lead to inaccurate estimation of some core parameters of the two-compartment model. Second, the PPK model in this study was established based on data from only 138 patients in the test set. Due to small sample size, the poor stability of FOCE-I parameter estimation resulted in inaccurate prediction of individual concentration and limitation in evaluating the nonlinear effects of covariates. Third, the P30 value of the PPK model in the paper is only 53.33%, which is much lower than LightGBM’s 89.13%. This confirms that its error model cannot adapt to the actual error distribution of the data—predictive errors of a large number of samples exceed ±30%, ultimately increasing RMSE and MAE. The poor fitting performance of the PPK model is the result of superimposed multiple limitations, and these limitations are further amplified in the complex pathological context of patients with liver disease. In contrast, LightGBM compensates for the shortcomings of the PPK model through features such as “no structural assumptions, nonlinear learning, and adaptive error minimization,” ultimately achieving significantly superior predictive performance. In the future, a large-sample size of the PPK model is needed and concentration-time curve data under non-steady-state conditions should be included, thereby comparing the predictive performance of PPK and machine learning models more comprehensively and accurately.

Conventional TEIC TDM applies generalized trough targets, overlooking the physiological and pathological differences among patients. Our prediction model provides real time pharmacokinetic forecasts, enabling personalized dose adjustments that keep concentrations therapeutic, accommodate patient variability. The predicted TEIC concentrations generated by our model can inform clinical practice by enabling precise dosing adjustments tailored to individual patient needs. For instance, when a patient’s predicted concentration falls below the therapeutic threshold, clinicians can proactively increase the TEIC dose to ensure effective treatment, thereby minimizing the risk of therapeutic failure. Conversely, if the predicted concentration exceeds safe limits, clinicians can reduce the dose to prevent potential toxicity, particularly in patients with compromised liver function who may experience altered pharmacokinetics (Pea, 2020; Zhang et al., 2024). This proactive approach aligns with the principles of precision medicine, which emphasize individualized treatment plans based on patient-specific data.

Our study provides a key strength through its large, real-world sample of TEIC pharmacokinetic data, comprising 646 liver disease patients across diverse etiologies. This heterogeneity encompassing varying disease subtypes, severities, and comorbidities accurately reflects clinical complexity. Such comprehensive data establishes a robust foundation for developing individualized dosing models, significantly enhancing the clinical applicability and translational potential of our findings. Incorporating this model into the TDM workflow could enhance its utility in clinical settings. By integrating the model’s predictions into routine TDM practices, clinicians can obtain real-time pharmacokinetic forecasts that guide dosing adjustments. For example, prior to sampling for TDM, the model could be employed to predict steady-state concentrations based on the patient’s clinical parameters and previous dosing history. This predictive capability allows for timely interventions, optimizing therapeutic outcomes while minimizing adverse effects (Abdul-Aziz et al., 2020; Mo et al., 2022).

Liver diseases include a wide spectrum of etiologies-such as hepatitis disease, ALD, and NASH-each with distinct pathophysiology that may alter TEIC pharmacokinetics (European Association for the Study of the Liver, 2017; Singal et al., 2018; Morgan et al., 2008). Our cohort mainly comprised hepatitis disease (28.16%), liver cirrhosis (20.61%), and FLD (13.64%), with few ALD cases (2.03%). This reflects China’s epidemiology but differs from Western populations where ALD and NASH are more common (GBD, 2017 Causes of Death Collaborators, 2018; Wang et al., 2014). In addition, the Child-Pugh classification (details of the classification are shown in the table below) was used to comprehensively assess liver function (Child-Pugh Class A: 38.90%; Class B: 44.27%; Class C: 16.84%) to better understand the liver function status of patients in this cohort.

This study has limitations. First, while our model was validated internally using 10-fold cross-validation, it was developed using data from a single center in China. This raises concerns about its external validity and generalizability. For instance, etiological diversity (such as diverse liver diseases) might influence model generalizability. Furthermore, ethnic differences in genetic polymorphisms, body composition, and other clinical factors can notably influence drug metabolism and CL (Stevens et al., 2011; Giacomini et al., 2010). Therefore, external validation is crucial. We are actively planning a prospective, multicenter study to collect data from diverse etiological and ethnic populations. This future work will be essential to confirm the model’s robustness and recalibrate it if necessary, ensuring its broader clinical applicability (Steyerberg and Vergouwe, 2014). Additionally, as TEIC is mainly renally eliminated, patients with renal impairment may show pharmacokinetic changes dominated by renal rather than hepatic factors. Although eGFR and urea were included as predictors, we did not perform a subgroup analysis stratified by renal function, which may limit model accuracy in patients with severe renal dysfunction. Further studies should include stratified analyses by renal function to clarify the relative roles of hepatic and renal covariates and support dosing optimization in patients with combined liver and kidney impairment. Second, observational data inherently contain missing values. Some variables with excessively high missing rates (such as LDL-C, TC, and TG) were excluded, which might have overlooked potential confounders, such as lipid metabolism’s impact on drug distribution. Third, the observational study design cannot establish causal inference between variables. Therefore, prospective trials should verify clinical benefits of model-guided dose adjustment, specifically improved efficacy and reduced toxicity. Last, this study did not explicitly model the internal correlation among the subjects, which is a methodological limitation. However, given the low proportion of repeated measurements (6.2%) and the dynamic changes in clinical features, we believe that the impact of this limitation on the model’s predictive performance is relatively limited. In the future, in larger-scale longitudinal datasets, we will explore mixed-effect models or sequence models (such as LSTM) to further verify the robustness of the results.

For future research, we plan to expand cohort diversity through multi-center recruitment to enhance model generalizability; second, to incorporate granular clinical indicators such as ascites depth and staging of HRS to improve predictions in severe phenotypes; third, to integrate physiologically based pharmacokinetic modeling with machine learning frameworks to augment interpretability and provide mechanistic insights for dose individualization; fourth, we plan to construct a PPK model with larger sample size and include concentration-time curve data under non-steady-state conditions, to comprehensively compare the predictive performance between PPK and machine learning models; last, future research should focus on validating the model’s predictions in diverse clinical settings and exploring its integration into automated TDM systems. Such advancements could notably enhance the safety and efficacy of TEIC therapy in patients with liver disease, ultimately improving patient outcomes.

In conclusion, this study developed the first machine learning model predicting TEIC plasma concentrations in liver disease patients using real-world data. Beyond established covariates (Daily dose, ALB, eGFR, PLT, urea), we newly identified five clinical variables (HGB, AST, IBIL, TBIL, and DBIL) as mechanistic predictors of TEIC exposure in liver disease patients. With robust predictive performance (R^2^ = 0.80), this model addresses the unmet need for individualized antibiotic dosing in current guidelines, providing a tool to advance precision antimicrobial therapy in this population.

## Data Availability

The raw data supporting the conclusions of this article will be made available by the authors, without undue reservation.
