# Supplementary material for: Machine learning-based prediction model for teicoplanin plasma concentrations in adults with liver disease using real-world data
Source: Front Pharmacol. 2025 Dec 5;16:1703976. doi: 10.3389/fphar.2025.1703976 (PMC12715011; doi:10.3389/fphar.2025.1703976)
Supplement: Supplementary file 1 [file DataSheet1.pdf]

Supplementary Material

Appendix 1. Derived feature formulas..2

Appendix 2. Model development principles..3

Appendix 3. Population pharmacokinetic model development..........10

Appendix 4. Population pharmacokinetic model evaluation.............12

Supplementary Tables 15

Supplementary Figures 23

Appendix 1. Derived feature formulas

The body mass index (BMI) formula is as follow:

$$\text{BMI}=\frac{W}{H^2}$$

where W is weight in kilograms, and H is height in meters. Because BMI is reported in kg·m^{-2}, all heights recorded in centimeters were converted to meters before calculation ($H=\frac{Height}{100}$).

The estimated glomerular filtration rate (eGFR) was calculated using the Chronic Kidney Disease Epidemiology Collaboration (CKD-EPI) creatinine equation (2021). In this study, Scr measurements are expressed in µmol/L. Therefore, to ensure clear and accurate results, a conversion factor (88.4) has been incorporated into the formula. The specific formula we used is as follows:

$$\text{eGFR (mL/min/1.73 } m^2\text{)} = 142\times\left(\frac{Scr}{88.4}\Big/A\right)^{B}\times(0.9938)^{Age}\times \text{C}$$

For women with a plasma creatinine/88.4 ≤0.7: A = 0.7, B= -0.241, C = 1.012;

For women with a plasma creatinine/88.4 >0.7: A = 0.7, B= -1.2, C = 1.012;

For men with a plasma creatinine/88.4 ≤0.9: A = 0.9, B= -0.302, C = 1;

For men with a plasma creatinine/88.4 >0.9: A = 0.9, B= -1.2, C = 1.

Appendix 2. Model development principles

1. LightGBM

LightGBM (Light Gradient Boosting Machine), introduced by Ke et al. (2017), offers an efficient and effective implementation of gradient boosting algorithms. This approach incorporates two key innovations: Gradient-based One-Side Sampling (GOSS) and Exclusive Feature Bundling (EFB). GOSS enhances traditional gradient boosting methods by preferentially selecting training instances which exhibit large gradients, thereby accelerating the learning process while reducing computational complexity. EFB is a technique designed to consolidate sparse features - those that predominantly take on zero values - and mutually exclusive features, such as one-hot encoded representations of categorical variables. Empirical evaluations across multiple public datasets demonstrate that LightGBM achieves comparable predictive accuracy while accelerating the training process of conventional Gradient Boosted Decision Trees (GBDT) by over 20-fold. This study employs the lightgbm software package to implement the LightGBM algorithm.

2. CatBoost

CatBoost (Categorical Boosting), proposed by Prokhorenkova et al. (2018), is a GBDT algorithm specifically optimized for categorical features. It implements a parameter-efficient framework with high accuracy by utilizing symmetric decision trees (oblivious trees) as base learners, primarily addressing the challenge of efficient and principled processing of categorical features. Additionally, CatBoost resolves gradient bias and prediction shift problems inherent in conventional gradient boosting methods,

thereby reducing overfitting and enhancing algorithmic accuracy and generalization capability. This study implements CatBoost using the catboost package.

3. XGBoost

XGBoost (extreme Gradient Boosting) is an efficient gradient boosting decision tree algorithm (Chen and Guestrin, 2016). It enhances the original GBDT framework, significantly improving model performance. As a forward stagewise additive model, its core methodology employs an ensemble approach - the boosting paradigm - which combines multiple weak learners into a single strong learner. Specifically, multiple trees collaboratively make predictions. Each tree predicts the residual (the difference between the target value and the sum of predictions from all previous trees). The final prediction is obtained by aggregating the results from all trees, thereby improving overall model performance. XGBoost consists of multiple CART (Classification And Regression Tree) base learners, enabling it to handle both classification and regression tasks. This study implements XGBoost using the xgboost package.

4. LinearRegression

LinearRegression is a regression analysis methodology that models the relationship between one or more independent variables and a dependent variable by minimizing the least squares function, known as the linear regression equation (Maulud and Abdulazeez, 2020). This approach establishes a linear relational model between independent and dependent variables through minimization of the sum of squared residuals (ordinary least squares method), thereby identifying the optimal linear fitting line to predict values of the dependent variable. The model architecture exhibits

inherent simplicity with straightforward implementation and interpretation, rendering it suitable for linearly-related data; moreover, its parameters possess explicit physical interpretations which facilitate analysis of the influence magnitude exerted by independent variables on the dependent variable. LinearRegression is categorized into univariate (single independent variable) and multivariate (multiple independent variables) subtypes. This study employs the linearregression module from the scikit-learn library.

5. SVM

SVM (Support Vector Machine) is a classical supervised learning algorithm for binary and multi-class classification tasks (Cortes and Vapnik, 1995). Its core principle involves solving an optimization problem framed as a convex quadratic programming problem to identify the optimal hyperplane. This formulation simultaneously minimizes model complexity while imposing constraints on training sample misclassification. The optimization problem is solvable via the Lagrange multiplier method. For linearly non-separable cases, SVMs employ kernel functions to map input features into higher-dimensional spaces, thereby transforming intrinsically non-separable data into linearly separable representations. Capable of performing linear/non-linear classification, regression, and outlier detection, SVM stands among the most prevalent models in machine learning, particularly well-suited for classifying complex small-to-medium-sized datasets. This study implements SVM using the sklearn package in Python.

6. TabPFN

TabPFN (Tabular Prior-data Fitted Network), proposed by Hollmann et al. (2025),

is an efficient neural architecture specifically designed for few-shot learning tasks on tabular data. This pre-trained Transformer performs supervised classification on small-scale tabular datasets in under one second without hyperparameter tuning, demonstrating competitiveness with state-of-the-art classification methods. TabPFN executes in-context learning (ICL) by leveraging labeled examples (x, f(x)) provided in the input sequence for prediction without requiring parameter updates. The model entirely encapsulates its functionality within network weights that accept training and test samples as set-valued inputs, generating predictions for entire test sets in a single forward pass. As a Prior-Data Fitted Network (PFN), TabPFN undergoes one-time offline training to approximate Bayesian inference on synthetic datasets drawn from its prior, which incorporates principles of causal inference through a large space of structural causal models with a bias toward simple structures. This study implements TabPFN using its open-source code repository.

7. TransTab

TransTab (Transferable Tabular Transformer), a flexible and transferable framework proposed by Wang and Sun, (2022), facilitates training and prediction on tabular data. Designed to streamline complex modeling procedures and enable cross-table learning, it enhances model generalization capabilities. This framework leverages pre-training and contrastive learning principles to optimize tabular data processing efficiency. TransTab allows seamless loading and processing of heterogeneous tabular datasets from diverse sources, utilizing pre-trained models for transfer learning to adapt to new tabular tasks. Additionally, it supports direct embedding vector encoding of

tabular data for downstream analytical applications. This study implements TransTab using PyTorch.

8. TabNet

TabNet (Attentive Interpretable Tabular Learning), proposed by Google Cloud AI in 2020, is a neural architecture specifically engineered for tabular data (Arik and Pfister, 2020). It synergistically integrates advantages of tree-based methods (interpretability and sparse feature selection) with strengths of deep neural networks (representation learning and end-to-end training). The model employs a sequential attention mechanism (analogous to additive models) to perform instance-wise feature selection, while its encoder-decoder framework enables self-supervised learning. This dual approach effectively combines the interpretability of tree models with the representational capacity of DNNs, yielding global feature selection capabilities that achieve superior performance on tabular datasets. This study implements TabNet using the pytorch_tabnet package.

9. DT

DT (Decision Trees), originating in 1966, constitute a fundamental machine learning algorithm that constructs a tree-based structure through recursive partitioning of datasets to perform classification and regression tasks (Freund and Schapire, 1997). Each node represents a feature, edges denote splitting criteria based on feature values, and leaf nodes correspond to prediction outcomes. The algorithm's core mechanism optimizes feature and splitting threshold selection at each node to maximize classification accuracy or minimize regression error. DT offers exceptional

interpretability and efficiently handles diverse data types, consequently seeing extensive application in data mining and machine learning. This study implements decision trees using the DecisionTreeRegressor module.

10. RF

RF (Random Forest), introduced by Leo Breiman in 2001, constitutes an ensemble learning algorithm that enhances prediction accuracy and robustness through constructing numerous decision trees for classification or regression tasks and aggregating their outcomes (Breiman, 2001). The stochastic nature of RF manifests in two critical aspects: firstly, bootstrap sampling randomly selects subsets of data points from the original training dataset during individual tree construction, generating distinct data subsets; secondly, at each split node within every tree, optimal partitioning is determined by evaluating only a randomly selected subset of features rather than all candidate features. This study employs the Random Forest Regressor implementation.

Reference

Arik, S. O., and Pfister, T. (2020). TabNet: Attentive interpretable tabular learning. doi: 10.48550/arXiv.1908.07442

Breiman, L. (2001). Random forests. *Machine Learning* 45, 5–32. doi: 10.1023/A:1010933404324

Chen, T., and Guestrin, C. (2016). XGBoost: A scalable tree boosting system., in *Proceedings of the 22nd ACM SIGKDD International Conference on Knowledge Discovery and Data Mining*, (New York, NY, USA: Association for Computing Machinery), 785–794. doi: 10.1145/2939672.2939785

Cortes, C., and Vapnik, V. (1995). Support-vector networks. *Mach Learn* 20, 273–297. doi: 10.1007/BF00994018

Freund, Y., and Schapire, R. E. (1997). A decision-theoretic generalization of on-line learning and an application to boosting. *Journal of Computer and System Sciences* 55, 119–139. doi: 10.1006/jcss.1997.1504

Hollmann, N., Müller, S., Purucker, L., Krishnakumar, A., Körfer, M., Hoo, S. B., et al. (2025). Accurate predictions on small data with a tabular foundation model. *Nature* 637, 319–326. doi: 10.1038/s41586-024-08328-6

Ke, G., Meng, Q., Finley, T., Wang, T., Chen, W., Ma, W., et al. (2017). LightGBM: A highly efficient gradient boosting decision tree., in *Proceedings of the 31st International Conference on Neural Information Processing Systems*, (Red Hook, NY, USA: Curran Associates Inc.), 3149–3157.

Maulud, D., and Abdulazeez, A. M. (2020). A review on linear regression comprehensive in machine learning. *Journal of Applied Science and Technology Trends* 1, 140–147. doi: 10.38094/jastt1457

Prokhorenkova, L., Gusev, G., Vorobev, A., Dorogush, A. V., and Gulin, A. (2018). CatBoost: Unbiased boosting with categorical features., in *Proceedings of the 32nd International Conference on Neural Information Processing Systems*, (Red Hook, NY, USA: Curran Associates Inc.), 6639–6649.

Wang, Z., and Sun, J. (2022). TransTab: Learning transferable tabular transformers across tables. Available at: https://openreview.net/forum?id=A1yGs_SWiIi (Accessed July 21, 2025).

Appendix 3. Population pharmacokinetic model development

Model development

In this study, a nonlinear mixed effects model (NONMEM, version 7.3.0) was used for the analysis of population pharmacokinetic (PPK), the establishment and validation of the model was achieved by Pirana (version 2.9.0). The PPK model is constructed based on the same dataset of the machine learning models. Apply a one-compartment model (ADVAN1 TRANS2) with first-order conditional estimation of inter-individual and intra-individual variability interactions (FOCE-I) to estimate clearance (CL/F) and volume of distribution (V/F). The inter-individual variation (IIV) was described by an exponential model (Equation 1), and the associative error (Equation 2) was used to fit the intra-individual variation.

$$P_{ij}=P_{tv,j}\cdot e^{\eta_i} \quad (1)$$

$$Y=F\cdot(1+\varepsilon_1)+\varepsilon_2 \quad (2)$$

In Equation 1, P_{ij} is the individual parameter values, $P_{tv,j}$ is the typical values of population parameters, while η_i denotes the random effect of each individual that follows a normal distribution with mean 0 and variance ω^2. In Equation 2, Y and F indicate the observed and predicted values of concentrations. ε_1 and ε_2 are the proportional error and summed error, obeying a normal distribution with a mean of 0 and variances of σ_1^2 and σ_2^2, respectively.

Covariate filtering

Constructed a comprehensive PPK model for TEIC by conducting covariate screening using forward inclusion method. If the included covariates reduce the

objective function value (OFV) by more than 6.63 (P <0.01), it is considered statistically significant and retained in the model. In backward exclusion, if removing a covariate increases the OFV by more than 10.83 (P <0.001), that variable is deemed significant. The final model was derived from the comprehensive model via backward exclusion. In the analysis, continuous variables were evaluated by Equation 3, while categorical variables were evaluated using Equation 4.

$$P_{ij}=P_{tv,j}\cdot(1\text{-}\theta_j\cdot(COV\text{-}COV_{med}))\cdot e^{\eta_i} \tag{3}$$

$$P_{ij}=P_{tv,j}\cdot(1\text{-}\theta_j\cdot COV)\cdot e^{\eta_i} \tag{4}$$

Therein, COV denotes covariates, COV_{med} represents the median of continuous covariates, and θ_j is the variable used to adjust the jth PK parameter. For sex, COV = 0 indicates males and COV = 1 indicates females. For comorbidity, COV = 0 indicates non-existence, and COV = 1 indicates existence.

Model evaluation

To evaluate the stability and predictability of the final model, relative standard error (RSE), goodness of fit (GOF) plot, and bootstrap method were used for validation. The GOF plots were produced using GraphPad Prism (version 10.1.2). The software package Perl-Seaks-NONMEM (PsN) (version 4.9.0) was used for bootstrap with 1000 repeated samples of the dataset, evaluating the accuracy of pharmacokinetic parameters by comparing the median and 95% confidence interval of raw data and results. To further assess the predictive performance of the PPK model, simulation was applied based on the test set, to predict the concentrations of teicoplanin and calculate RMSE, R^2, MAE, and the proportion of predictions within ±30% (P30).

Appendix 4. Population pharmacokinetic model evaluation

PPK model establishment

After covariate screening, we obtained the final model (Equation 5 and Equation 6). The detailed parameters of PPK were shown in Table A1. Population typical values of CL/F and V/F were 0.77 L/h and 51.6 L, respectively. The RSE values were below 30%, indicating a reasonable range of the parameters in the final model. The results showed that urea levels have a negatively notable effect on the CL/F of TEIC, while V/F remained unaffected by covariates.

$$CL/F\ (L{\cdot}\mathrm{h}^{-1})=0.77\times[1-0.013\times(Urea-11.3)] \quad (5)$$

$$V/F\ (L)=51.6 \quad (6)$$

Table A1. Population pharmacokinetic parameters and bootstrap results for teicoplanin

Parameters	Basic Model		Final Model			Bootstrap (n=1000)	
	Estimate	RSE (%)	Estimate	RSE (%)	IIV (%)	Median	95% CI
CL/F ($L{\cdot}h^{-1}$)	0.741	7	0.77	5	0.139	0.765	0.687-0.847
V/F (L)	52	17	51.6	7	0.307	52.23	44.52-64.70
$\theta_{CL\text{-}Urea}$	-	-	0.013	21	-	0.013	0.0019-0.0153
PRO (%)	0.0597	-	0.0689	-	-	0.0591	0.0241-0.0866
ADD (%)	6.17	-	2.82	-	-	3.60	0.134-20.408

Model evaluation

Figure A1 showed the GOF plots for the final model, including population predicted concentrations versus observed concentrations (Figure A1-A), individual predicted concentrations versus observed concentrations (Figure A1-B), conditional weighted residuals (CWRES) versus individual predicted concentrations (Figure A1-

C), and CWRES versus time after first dose (Figure A1-D). Among these, a good linear relationship existed between observed and predicted concentrations and the majority of CWRES falled within ±2, indicating that overall fitting performance was satisfactory. Table A1 also listed the outcomes of the bootstrap method. The method succeeded 832 out of 1000 validations, with a success rate of 83.2%. The medians of the results from bootstrap datasets were similar to the estimate values of the final model, and the 95% confidence interval also encompassed the estimate values in final model. In summary, these results concluded good stability and predictability of the final model for teicoplanin.

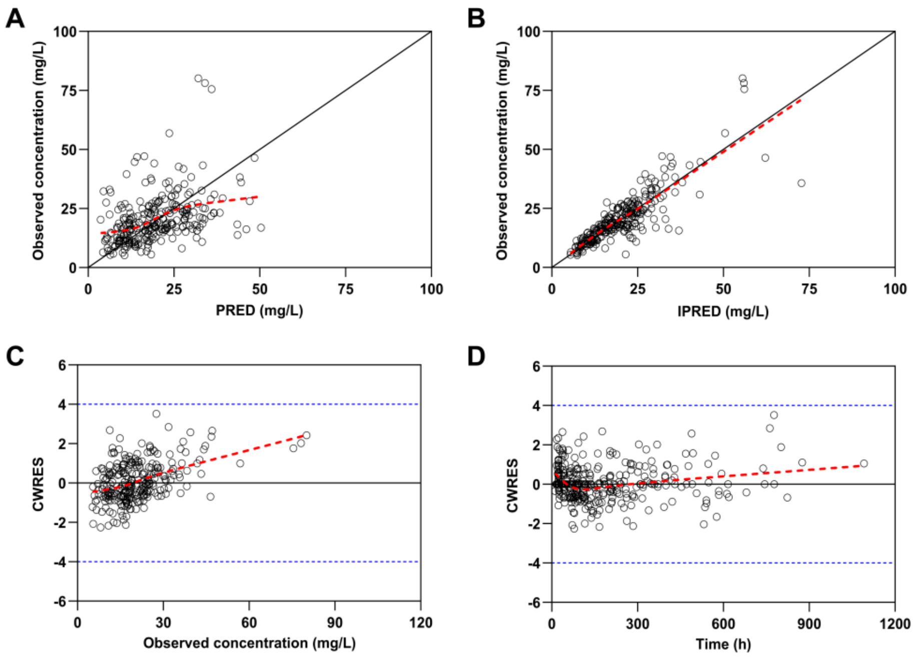

Figure A1 Plots of goodness-of-fit of the final model. (**A**) Population predicted concentrations versus observed concentrations. (**B**) Individual predicted concentrations versus observed concentrations. (**C**) Conditional weighted residuals versus individual predicted concentrations. (**D**) Conditional weighted residuals versus time after first dose. The solid black lines are the reference lines and the dotted red lines are the locally weighted regression trend lines.

Predictive performance of the PPK model

The predictive performance metrics of the final PPK model are presented in Table

A2. It shows that the PPK model achieved a R^2 of 0.68, a RMSE of 22.60, a MAE of 16.11, and a P30 of 53.33%.

Table A2. Predictive performance metrics of the final PPK model for TEIC

RMSE	R^2	MAE	P30 (%)
22.60	0.68	16.11	53.33

Abbreviations: RMSE, root mean square error; R^2, coefficient of determination; MAE, mean absolute error; P30, proportion of predictions within ±30% of the observed value.

Supplementary Tables

Supplementary Table S1. Accuracy and precision of the HPLC method for teicoplanin quantification in human serum

LLOQ and QC Standards (μg/mL)	Accuracy (%)	Intra-day precision (RSD%, n = 5)	Inter-day precision (RSD%, n = 3)
3.125	101.72	1.38	2.85
3.70	104.09	2.82	3.55
11.11	107.55	0.27	6.35
33.33	107.63	1.17	4.68

Note: The HPLC method demonstrated acceptable accuracy (90-110%) and precision (RSD <10%) across all concentration levels, meeting FDA and EMA bioanalytical validation guidelines.

Abbreviations: LLOQ, lower limit of quantitation; QC, quality control; RSD, relative standard deviation.

Supplementary Table S2. Sensitivity analysis

Method	Model	RMSE	R^2	MAE
RF	DT	3.43 ± 0.34	0.67 ± 0.04	2.81 ± 0.29
Bayes	DT	3.43 ± 0.34	0.67 ± 0.04	2.81 ± 0.29
KNN	DT	3.43 ± 0.34	0.67 ± 0.04	2.81 ± 0.29
Mean	DT	3.43 ± 0.34	0.67 ± 0.04	2.81 ± 0.29
Median	DT	3.43 ± 0.34	0.67 ± 0.04	2.81 ± 0.29
MICE	DT	3.43 ± 0.34	0.67 ± 0.04	2.81 ± 0.29
RF	TabPFN	3.37 ± 0.34	0.68 ± 0.04	2.72 ± 0.27
Bayes	TabPFN	3.37 ± 0.32	0.68 ± 0.04	2.72 ± 0.26
KNN	TabPFN	3.38 ± 0.36	0.68 ± 0.04	2.73 ± 0.28
Mean	TabPFN	3.37 ± 0.32	0.68 ± 0.04	2.72 ± 0.25
Median	TabPFN	3.37 ± 0.33	0.68 ± 0.04	2.72 ± 0.26
MICE	TabPFN	3.36 ± 0.32	0.68 ± 0.04	2.72 ± 0.26
RF	SVM	4.01 ± 0.55	0.55 ± 0.05	3.08 ± 0.37
Bayes	SVM	3.97 ± 0.55	0.56 ± 0.06	3.03 ± 0.37
KNN	SVM	4.00 ± 0.57	0.56 ± 0.05	3.05 ± 0.37

Mean	SVM	3.98 ± 0.56	0.56 ± 0.05	3.03 ± 0.35
Median	SVM	3.98 ± 0.57	0.56 ± 0.06	3.03 ± 0.36
MICE	SVM	3.98 ± 0.56	0.56 ± 0.06	3.03 ± 0.37
RF	LinearRegression	3.56 ± 0.40	0.65 ± 0.04	2.85 ± 0.28
Bayes	LinearRegression	3.57 ± 0.40	0.64 ± 0.05	2.84 ± 0.29
KNN	LinearRegression	3.56 ± 0.41	0.65 ± 0.05	2.84 ± 0.29
Mean	LinearRegression	3.56 ± 0.40	0.65 ± 0.05	2.85 ± 0.28
Median	LinearRegression	3.55 ± 0.40	0.65 ± 0.05	2.83 ± 0.29
MICE	LinearRegression	3.56 ± 0.40	0.65 ± 0.05	2.84 ± 0.29
RF	CatBoost	3.58 ± 0.53	0.65 ± 0.03	2.83 ± 0.33
Bayes	CatBoost	3.60 ± 0.53	0.64 ± 0.04	2.85 ± 0.33
KNN	CatBoost	3.59 ± 0.53	0.64 ± 0.03	2.84 ± 0.33
Mean	CatBoost	3.59 ± 0.54	0.64 ± 0.04	2.84 ± 0.33
Median	CatBoost	3.58 ± 0.53	0.65 ± 0.03	2.83 ± 0.33
MICE	CatBoost	3.60 ± 0.54	0.64 ± 0.04	2.84 ± 0.33
RF	TransTab	3.67 ± 0.43	0.62 ± 0.05	2.83 ± 0.23
Bayes	TransTab	3.67 ± 0.45	0.62 ± 0.05	2.82 ± 0.25
KNN	TransTab	3.66 ± 0.44	0.62 ± 0.05	2.81 ± 0.24
Mean	TransTab	3.67 ± 0.44	0.62 ± 0.05	2.82 ± 0.24
Median	TransTab	3.67 ± 0.44	0.62 ± 0.05	2.81 ± 0.24
MICE	TransTab	3.66 ± 0.44	0.62 ± 0.05	2.81 ± 0.25
RF	LightGBM	3.35 ± 0.37	0.69 ± 0.03	2.69 ± 0.27
Bayes	LightGBM	3.37 ± 0.36	0.68 ± 0.03	2.70 ± 0.26
KNN	LightGBM	3.35 ± 0.37	0.69 ± 0.03	2.69 ± 0.28
Mean	LightGBM	3.36 ± 0.37	0.69 ± 0.03	2.69 ± 0.28
Median	LightGBM	3.34 ± 0.37	0.69 ± 0.03	2.68 ± 0.27
MICE	LightGBM	3.37 ± 0.38	0.68 ± 0.03	2.70 ± 0.27
RF	XGBoost	3.82 ± 0.62	0.60 ± 0.05	2.98 ± 0.39
Bayes	XGBoost	3.88 ± 0.62	0.58 ± 0.05	3.01 ± 0.38
KNN	XGBoost	3.87 ± 0.62	0.59 ± 0.04	3.01 ± 0.38
Mean	XGBoost	3.89 ± 0.60	0.58 ± 0.04	3.01 ± 0.36
Median	XGBoost	3.87 ± 0.58	0.59 ± 0.04	3.00 ± 0.35
MICE	XGBoost	3.89 ± 0.62	0.58 ± 0.04	3.01 ± 0.36
RF	TabNet	3.72 ± 0.43	0.60 ± 0.09	2.90 ± 0.27
Bayes	TabNet	3.92 ± 0.55	0.56 ± 0.10	3.08 ± 0.26
KNN	TabNet	3.82 ± 0.50	0.58 ± 0.10	2.97 ± 0.28
Mean	TabNet	3.88 ± 0.45	0.57 ± 0.08	2.97 ± 0.23

Median	TabNet	3.99 ± 0.49	0.55 ± 0.07	3.06 ± 0.25
MICE	TabNet	3.96 ± 0.42	0.54 ± 0.14	3.07 ± 0.23
RF	RF	3.92 ± 0.59	0.58 ± 0.02	3.01 ± 0.36
Bayes	RF	3.92 ± 0.58	0.58 ± 0.03	3.02 ± 0.36
KNN	RF	3.91 ± 0.58	0.58 ± 0.03	3.02 ± 0.35
Mean	RF	3.89 ± 0.59	0.58 ± 0.03	2.99 ± 0.35
Median	RF	3.89 ± 0.59	0.58 ± 0.02	2.99 ± 0.35
MICE	RF	3.92 ± 0.60	0.58 ± 0.03	3.01 ± 0.37

Note: Performance comparison of ten machine learning models using six different imputation strategies (RF, Bayesian, KNN, mean, median, MICE). Values represent mean ± standard deviation from 10-fold cross-validation on the training set. RF imputation demonstrated consistently superior or equivalent performance across algorithms and was therefore selected as the primary imputation strategy.

Abbreviations: RF, random forest; Bayes, Bayesian; KNN, K-nearest neighbors; MICE, multiple imputation by chained equations; RMSE, root mean square error ; R^2, coefficient of determination; MAE, mean absolute error; DT, decision tree; TabPFN, tabular prior-data fitted network; SVM, support vector machine; CatBoost, categorical boosting; TransTab, transferable tabular transformer; LightGBM, light gradient boosting machine; XGBoost, extreme gradient boosting; TabNet, attentive interpretable tabular learning.

Table S3. The candidate parameters for ten-fold cross-validation of ten models

Algorithm	Parameter
LightGBM	param_grid = {<br>'learning_rate': [0.01, 0.03, 0.05, 0.08, 0.1, 0.3],<br>'max_depth': [3, 4, 5, 6],<br>}
CatBoost	param_grid = {

	'learning_rate': [0.01, 0.03, 0.05, 0.08, 0.1, 0.3],<br>'depth': [3, 4, 5, 6],<br>}
XGBoost	param_grid = {<br>'learning_rate': [0.01, 0.03, 0.05, 0.08, 0.1, 0.3],<br>'n_estimators': [100, 150, 200, 250, 300],<br>'max_depth': [3, 4, 5, 6],<br>}
LinearRegression	param_grid = {<br>'fit_intercept': [True, False],<br>'normalize': [True, False],<br>}
SVM	param_grid = {<br>'kernel': ['linear', 'poly', 'rbf', 'sigmoid'],<br>'gamma': [0.01, 0.1, 1, 10],<br>'C': [1e-2, 1e-1, 1, 10],<br>}
TabPFN	param_grid = {<br>'n_estimators': [8, 16, 32]<br>}
TransTab	param_grid = {<br>'hidden_dim': [8, 16, 32, 64],<br>'num_epoch': [20, 50, 100, 200, 500],<br>'lr': [1e-6, 1e-5, 1e-4, 1e-3, 1e-2, 1e-1],<br>}
TabNet	param_grid = {<br>'n_d': [8, 16, 32],<br>'n_a': [8, 16, 32],<br>'n_steps': [1, 3, 5],<br>'max_epochs': [10, 20, 40, 80, 150, 300],<br>'batch_size': [4, 8, 16, 32, 64],<br>'patience': [10, 20],<br>}
DT	param_grid = {<br>'min_samples_split': [2, 5, 10, 20],<br>'max_depth': [3, 4, 5, 6],<br>'max_features': ['log2', 'sqrt', 'auto', None]<br>}
RF	param_grid = {<br>'n_estimators': [100, 150, 200, 250],<br>'max_depth': [3, 4, 5, 6],<br>'max_features': ['log2', 'sqrt', 'auto', None]<br>}

Abbreviations: DT, decision tree; RF, random forest; XGBoost, extreme gradient boosting; LightGBM, light gradient boosting machine; CatBoost, categorical boosting; SVM, support vector machine; TabPFN, tabular prior-data fitted network; TransTab, transferable tabular transformer; TabNet, attentive interpretable tabular learning.

Table S4. The optimal parameters of ten models

Algorithm	Parameter
LightGBM	early_stop_round=20, learning_rate=0.01, max_depth=3, n_estimators=300,
CatBoost	iterations=500, early_stopping_rounds=10, learning_rate=0.01, depth=4
XGBoost	learning_rate=0.01, n_estimators=100, max_depth=4
LinearRegression	fit_intercept=True, normalize=False
SVM	kernel= 'linear', gamma= 0.01, C= 0.1
TabPFN	n_estimators=4
TransTab	hidden_dim=16, num_epoch=20, lr=1e-4
TabNet	n_d=8, n_a=8, n_steps=1, max_epochs=20, batch_size=8, patience=10
DT	max_depth= 3, max_features= None, min_samples_split= 2
RF	n_estimators=200, max_depth=4, max_features='log2'

Abbreviations: DT, decision tree; RF, random forest; XGBoost, extreme gradient boosting; LightGBM, light gradient boosting machine; CatBoost, categorical boosting; SVM, support vector machine; TabPFN, tabular prior-data fitted network; TransTab, transferable tabular transformer; TabNet, attentive interpretable tabular learning.

Supplementary Table S5. Feature importance rankings from four machine learning algorithms

RandomForest	LightGBM	XGBoost	CatBoost

Feature	Importance	Feature	Importance	Feature	Importance	Feature	Importance
daily dose	0.710536564	Urea	292	daily dose	0.879613757	daily dose	50.45461139
eGFR	0.046862482	eGFR	292	eGFR	0.01595697	Urea	6.43584728
Urea	0.045577587	ALB	265	ALB	0.013499423	ALB	5.812984791
ALB	0.036876284	AST	253	Urea	0.012948996	HGB	5.473318556
HGB	0.03038776	IBIL	253	HGB	0.012328841	eGFR	4.903524587
PLT	0.026034347	daily dose	249	IBIL	0.010215109	PLT	4.777902129
AST	0.024107803	PLT	240	PLT	0.009572187	AST	4.455976747
TBIL	0.02260175	HGB	219	DBIL	0.009532045	IBIL	4.388921448
IBIL	0.017862028	TBIL	156	AST	0.007080393	TBIL	3.722897839
DBIL	0.016158055	DBIL	152	TBIL	0.007048119	DBIL	3.47112346
BMI	0.013652247	BMI	114	Hypertension disease	0.006927124	BMI	2.66860788
Hypertension disease	0.002678936	Hypertension disease	32	Hepatitis disease	0.004318764	Hypertension disease	0.993314568
Circulation system disease	0.002528894	Circulation system disease	21	BMI	0.004230791	Hepatitis disease	0.935491204

Hepatitis disease	0.002428879	Hepatitis disease	20	Circulation system disease	0.004205306	Circulation system disease	0.911209141
Cirrhosis disease	0.001706381	Cirrhosis disease	5	Cirrhosis disease	0.002522151	Cirrhosis disease	0.594268976

Note: Importance scores were calculated independently for Random Forest (RF), LightGBM, XGBoost, and CatBoost. The final set of 10 core variables was determined by identifying features that appeared in the top 15 across all four models using intersection operations, ensuring robustness and reducing model-specific bias.

Abbreviations: CatBoost, categorical boosting; LightGBM, light gradient boosting machine; XGBoost, extreme gradient boosting; ALB, albumin; TBIL, total bilirubin; HGB, hemoglobin; eGFR, estimated glomerular filtration rate; PLT, platelet count; IBIL, indirect bilirubin; AST, aspartate aminotransferase; DBIL, direct bilirubin; BMI, body mass index.

Table S6. Statistical analysis of training set and test set

Category	Variable	Training set	Test set	*P* value
**Target variable**	TDM, median (IQR)	15.29 (11.83~19.37)	15.28 (12.24~19.10)	0.734
**TEIC daily dose**	Daily dose (g), median (IQR)	0.40 (0.40~0.60)	0.40 (0.40~0.60)	0.773
**Laboratory test**	AST (U/L), median (IQR)	42.00 (25.00~76.10)	46.80 (26.00~83.92)	0.711
	Urea (mmol/L), median (IQR)	10.70 (5.90~18.70)	10.75 (6.21~17.43)	0.962
	TBIL (μmol/L), median (IQR)	27.00 (10.60~115.40)	18.95 (9.00~97.46)	0.191
	ALB (g/L), median (IQR)	33.70 (30.30~37.00)	33.20 (29.62~37.18)	0.631
	DBIL (μmol/L), median (IQR)	18.20 (6.00~94.85)	13.45 (4.96~79.80)	0.207

eGFR (ml/min/1.73m^2), median (IQR)	67.89 (30.11~102.36)	61.65 (27.94~98.42)	0.462
PLT (10^9/L), median (IQR)	121.00 (58.50~241.50)	132.00 (70.25~224.75)	0.372
HGB (g/L), median (IQR)	76.00 (64.00~97.00)	76.00 (64.25~90.00)	0.522
IBIL (μmol/L), median (IQR)	7.70 (4.20~22.10)	5.95 (3.73~18.90)	0.229

Abbreviations: TDM, therapeutic drug monitoring; AST, aspartate aminotransferase; TBIL, total bilirubin; ALB, albumin; DBIL, direct bilirubin; eGFR, estimated glomerular filtration rate; PLT, platelet count; HGB, hemoglobin; IBIL, indirect bilirubin.

Table S7. Model performance on the test set

Algorithm	RMSE	R^2	MAE
LightGBM	2.90	0.80	2.34
CatBoost	3.40	0.73	2.62
XGBoost	3.65	0.69	2.70
LinearRegression	3.42	0.72	2.64
SVM	4.08	0.61	3.01
TabPFN	3.00	0.79	2.37
TransTab	3.30	0.74	2.53
TabNet	3.33	0.74	2.59
DT	3.12	0.77	2.56
RF	3.79	0.66	2.80

Abbreviations: RMSE, root mean square error; R^2, coefficient of determination; MAE, mean absolute error; DT, decision tree; RF, random forest; TransTab, transferable tabular transformer; XGBoost, extreme gradient boosting; LightGBM, light gradient boosting machine; CatBoost, categorical boosting; SVM, support vector machine; TabPFN, tabular prior-data fitted network; TabNet, attentive interpretable tabular learning.

Supplementary Figures

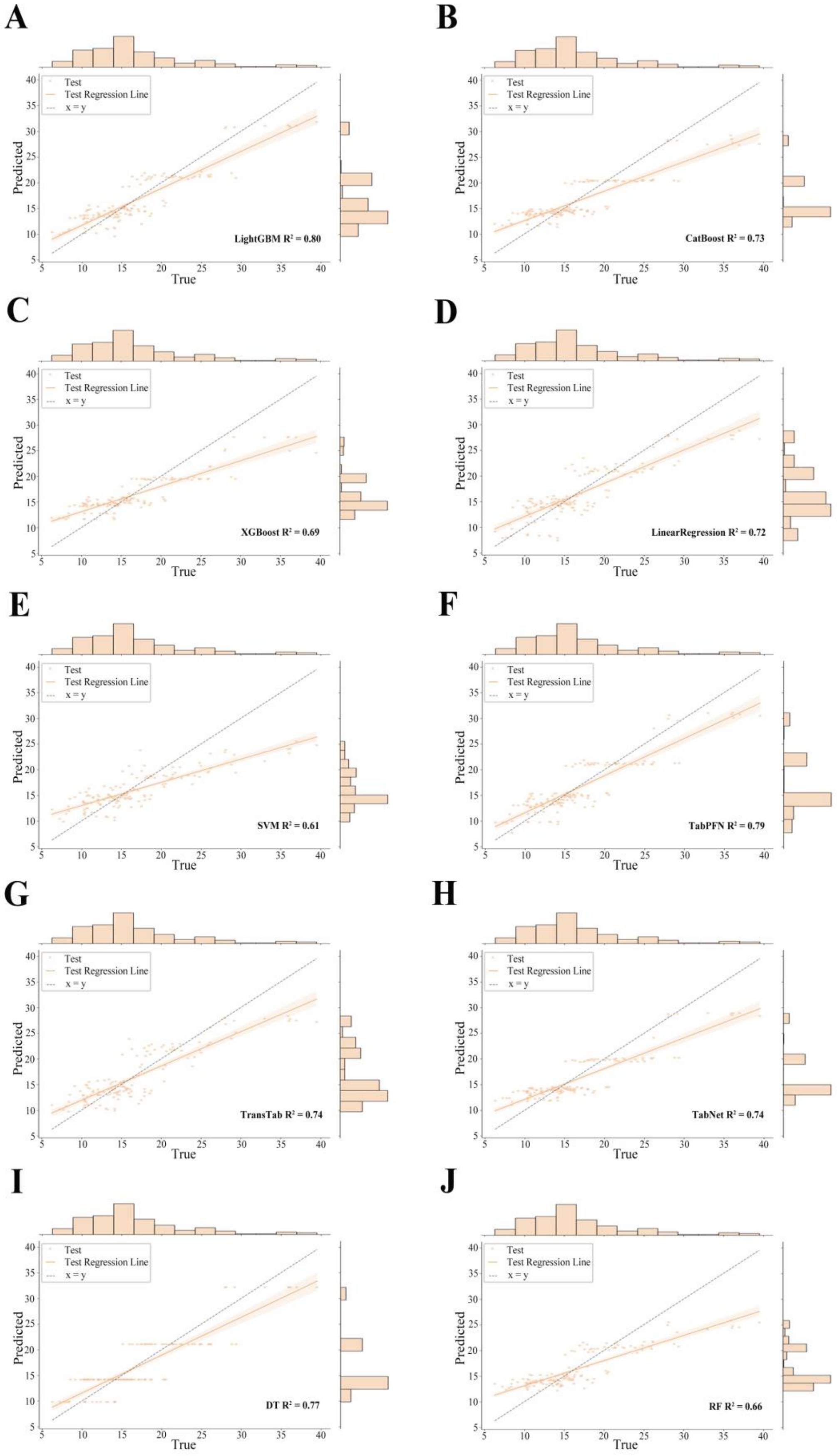

Figure S1. Predicted versus observed TEIC plasma concentration scatter plots for ten models.

Note: A) LightGBM, B) CatBoost, C) XGBoost, D) LinearRegression, E) SVM, F) TabPFN, G) TransTab, H) TabNet, I) DT, J) RF.Each panel represents a different machine learning algorithm. The diagonal line represents perfect prediction (y=x). R^2 values indicate model performance.

Abbreviations: R^2, coefficient of determination; DT, decision tree; RF, random forest; XGBoost, extreme gradient boosting; LightGBM, light gradient boosting machine; CatBoost, categorical boosting; SVM, support vector machine; TabPFN, tabular prior-data fitted network; TransTab, transferable tabular transformer; TabNet, attentive interpretable tabular learning.

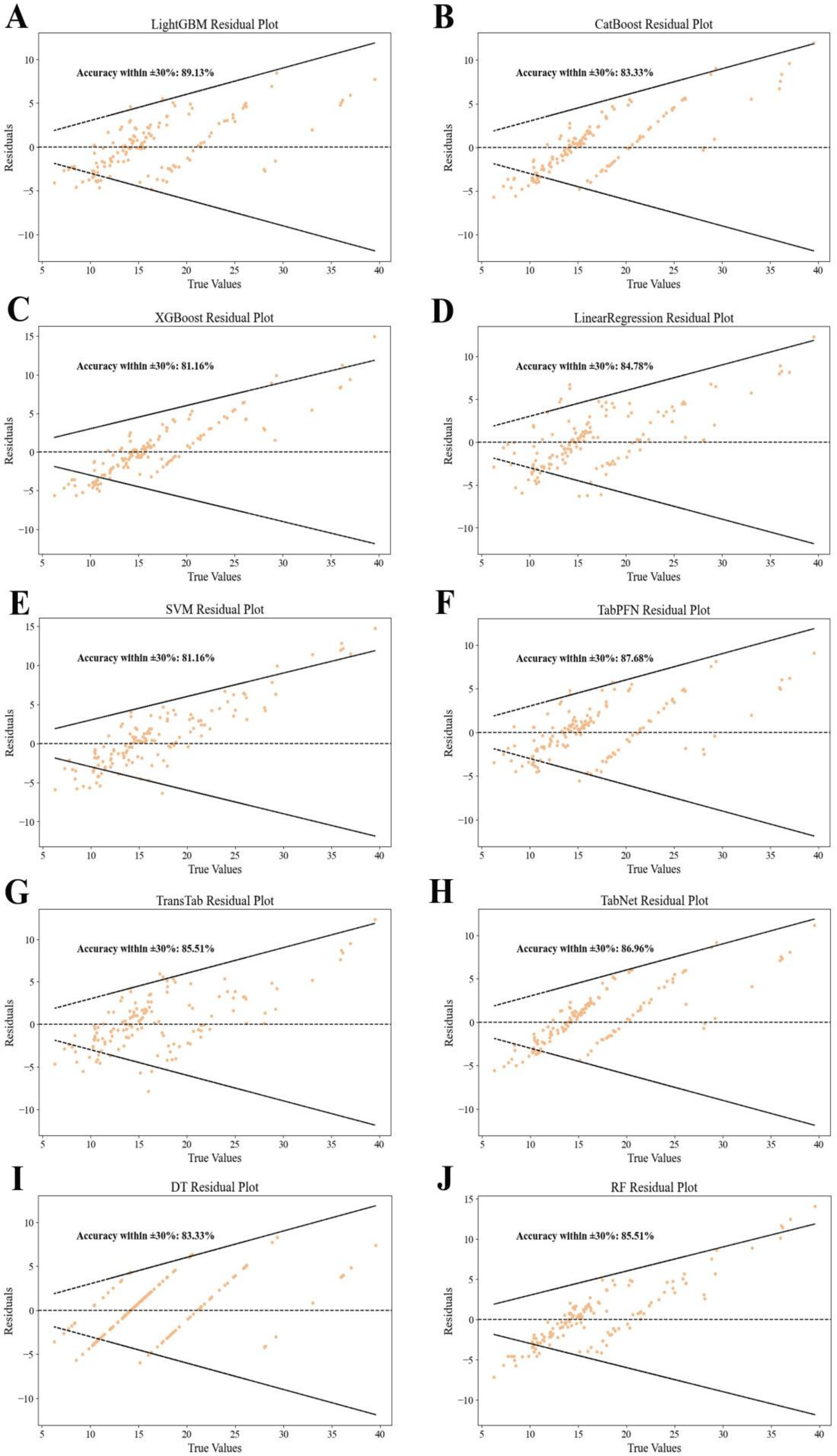
A
LightGBM Residual Plot
Accuracy within ±30%: 89.13%
B
CatBoost Residual Plot
Accuracy within ±30%: 83.33%
C
XGBoost Residual Plot
Accuracy within ±30%: 81.16%
D
LinearRegression Residual Plot
Accuracy within ±30%: 84.78%
E
SVM Residual Plot
Accuracy within ±30%: 81.16%
F
TabPFN Residual Plot
Accuracy within ±30%: 87.68%
G
TransTab Residual Plot
Accuracy within ±30%: 85.51%
H
TabNet Residual Plot
Accuracy within ±30%: 86.96%
I
DT Residual Plot
Accuracy within ±30%: 83.33%
J
RF Residual Plot
Accuracy within ±30%: 85.51%
Residuals
True Values

Figure S2. Residual plots showing prediction errors for ten models.

Note: A) LightGBM, B) CatBoost, C) XGBoost, D) LinearRegression, E) SVM, F) TabPFN, G) TransTab, H) TabNet, I) DT, J) RF. Each panel displays the distribution of prediction errors (observed - predicted values). The percentage indicates the proportion of predictions falling within ±30% of observed values.

Abbreviations: DT, decision tree; RF, random forest; XGBoost, extreme gradient boosting; LightGBM, light gradient boosting machine; CatBoost, categorical boosting; SVM, support vector machine; TabPFN, tabular prior-data fitted network; TransTab, transferable tabular transformer; TabNet, attentive interpretable tabular learning.

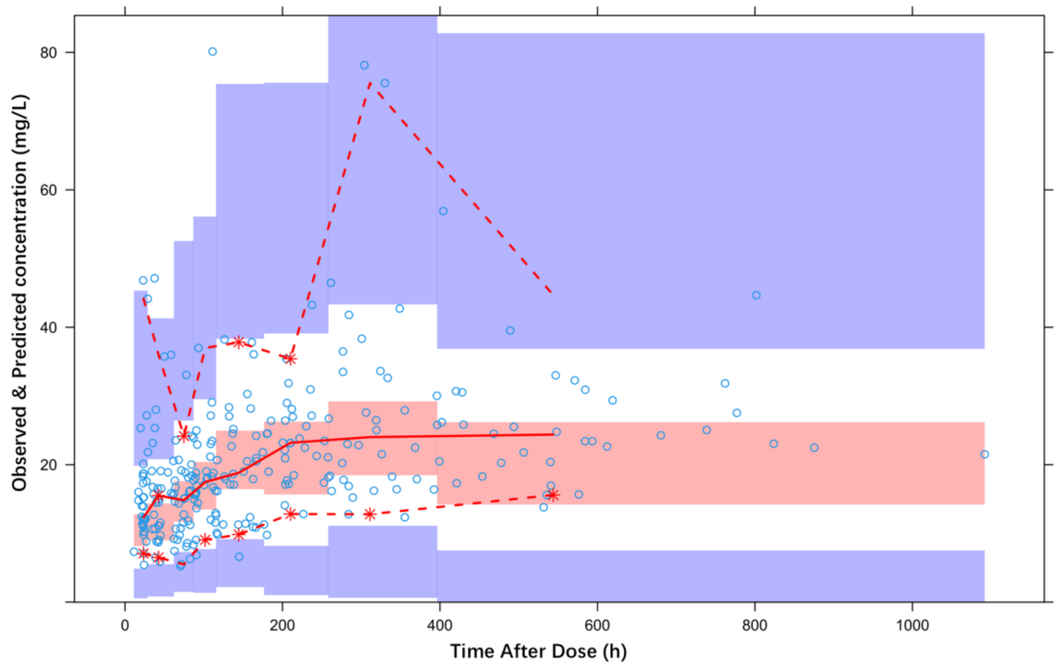

Figure S3. The visual predictive check (VPC) for the TEIC PPK model.

Blue dots: observed concentrations after dosing. The solid, lower-dashed and upper-dashed lines: 50%, 5%, and 95% percentiles of observations. Shaded regions: The red,

lower and upper blue shadows represent the 95% confidence intervals of the 50%, 5% and 95% percentiles of the predicted values.

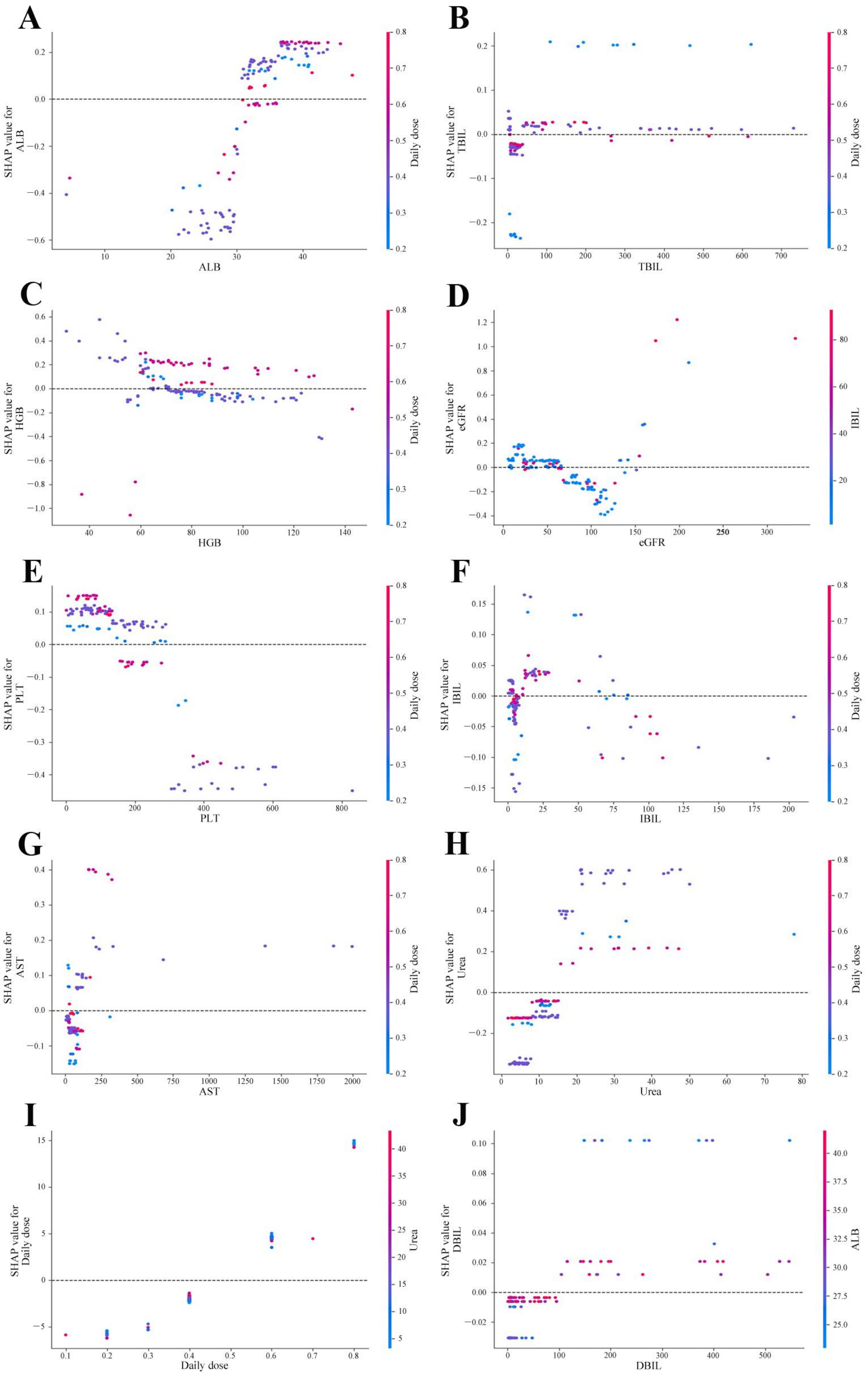

Figure S4. SHAP dependence plot based on the LightGBM model.

Note: A) ALB, B) TBIL, C) HGB, D) eGFR, E) PLT, F) IBIL, G) AST, H) urea, I) daily dose, J) DBIL. Color gradient (blue→red) denotes increasing feature values. Dashed lines indicate clinically significant thresholds.

Abbreviations: AST, aspartate aminotransferase; TBIL, total bilirubin; ALB, albumin; DBIL, direct bilirubin; eGFR, estimated glomerular filtration rate; PLT, platelet count; HGB, hemoglobin; IBIL, indirect bilirubin.

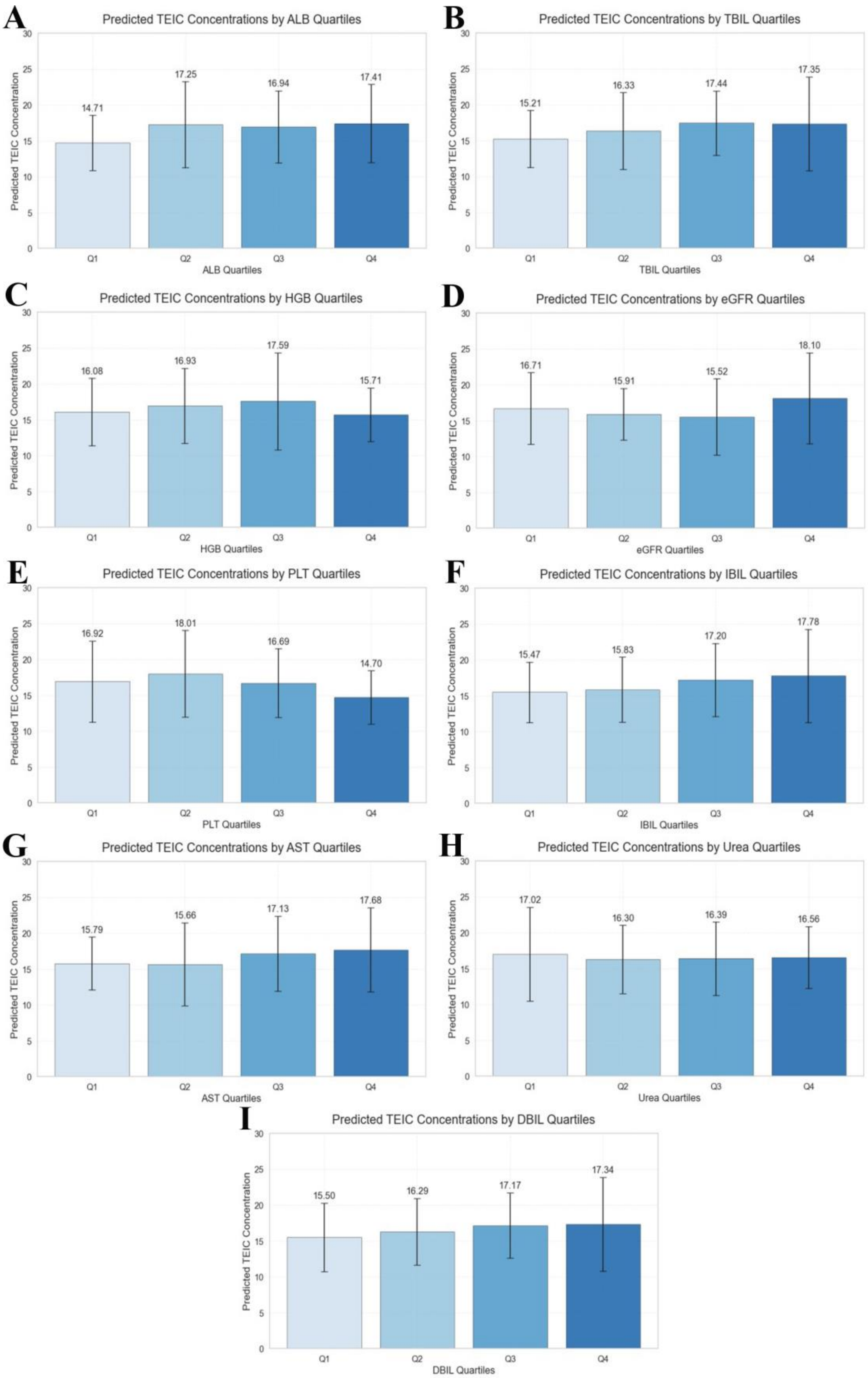

Supplementary Figure S5. Predicted TEIC concentrations across quartiles of continuous variables.

Note: Bars represent mean predicted TEIC concentrations for each quartile (Q1: 0-25%, Q2: 25%-50%, Q3: 50%-75%, Q4: 75%-100%) based on sample distribution. Error bars indicate standard deviation. Quartiles were defined to ensure equal sample sizes across groups (approximately 25% per quartile). A) ALB (albumin), B) TBIL (total bilirubin), C) HGB (hemoglobin), D) eGFR (estimated glomerular filtration rate), E) PLT (platelet count), F) IBIL (indirect bilirubin), G) AST (aspartate aminotransferase), H) Urea, I) DBIL (direct bilirubin).
